# Erythrocyte Glycolytic and Redox Metabolism Affects Muscle Oxygenation and Exercise Performance: A Randomized Double-Blind Crossover Study in Men

**DOI:** 10.1007/s40279-025-02279-2

**Published:** 2025-07-28

**Authors:** Panagiotis N. Chatzinikolaou, Nikos V. Margaritelis, Vassilis Paschalis, Anastasios A. Theodorou, Eleni Moushi, Ioannis S. Vrabas, Antonios Kyparos, Ioannis G. Fatouros, Angelo D’Alessandro, Michalis G. Nikolaidis

**Affiliations:** 1 Department of Physical Education and Sports Science at Serres, Aristotle University of Thessaloniki, Serres, Greece; 2 School of Physical Education and Sport Science, National and Kapodistrian University of Athens, Athens, Greece; 3 Department of Life Sciences, School of Sciences, European University Cyprus, Nicosia, Cyprus; 4 Department of Physical Education and Sport Sciences, University of Thessaly, Trikala, Greece; 5 Department of Biochemistry and Molecular Genetics, University of Colorado Anschutz Medical Campus, Aurora, CO USA

## Abstract

**Background:**

Erythrocytes are traditionally considered passive oxygen carriers, yet their energetic and redox metabolism plays a critical role in regulating oxygen kinetics.

**Objective:**

This study integrates experimental and computational data to provide a comprehensive analysis of erythrocyte metabolism in response to exercise-induced oxidative stress.

**Methods:**

The study consisted of three phases: in vivo, ex vivo, and computational. A total of 20 male participants underwent a randomized crossover experiment with two conditions: oxidative stress (eccentric contractions) and control. Oxidative stress was induced via leg eccentric contractions, and its effects on erythrocyte glycolytic and redox metabolism, arm muscle oxygenation, and arm exercise performance were evaluated. The study protocol was preregistered on the Open Science Framework (https://osf.io/ub6zs).

**Results:**

Eccentric contractions altered oxidative stress markers in erythrocytes (+ 22% F_2_-isoprostanes, + 28% protein carbonyls, − 20% glutathione). Oxidative stress increased erythrocyte glycolytic flux by + 53%, while arm exercise further increased glycolytic flux in both control (+ 200%) and oxidative stress (+ 86%) conditions. Exogenous hydrogen peroxide administration reduced glycolytic flux by − 48%. Stoichiometric analysis revealed that during acute exercise, erythrocytes produced 14.9% less ATP, NADPH, and 2,3-bisphosphoglycerate than their theoretical maximum, at the critical bioenergetic point. Oxidative stress decreased arm deoxygenated hemoglobin by − 7.4% during arm exercise and VO_2_peak by − 4% during arm exercise.

**Conclusion:**

In a comprehensive exercise study investigating mechanistic relationships in erythrocyte biology, we show that erythrocyte metabolism (1) responds dynamically to exercise, (2) becomes dysregulated under oxidative stress, and (3) may partly influence muscle oxygenation and performance.

**Supplementary Information:**

The online version contains supplementary material available at 10.1007/s40279-025-02279-2.

## Key Points

**Table:** 

The erythrocyte glycolytic and redox metabolism responds dynamically to exercise and possibly become dysregulated under oxidative stress.
The erythrocyte metabolism may partly determine muscle oxygenation and exercise performance.
We highlight the need to focus on erythrocyte biology, which may add an overlooked cell to the list of contributors to fatigue and deepen our understanding of endurance physiology.

## Introduction

The erythrocyte has a rich historical significance, extensively studied since the early 1900s [1–4], with its basic metabolism and functions among the first described [5–7]. It has been widely used as a cell model owing to distinct characteristics: absence of organelles, ease of collection, simple structure predominantly composed of hemoglobin (~ 97% dry mass), and exclusive reliance on glycolysis for ATP production [8, 9]. These features contributed to a misconception of erythrocytes as passive oxygen carriers [9].

In exercise physiology, improvements in muscle oxygen utilization are typically attributed to increased erythrocyte number or hemoglobin levels [10–12]. This is exemplified in studies showing that (1) erythrocyte and hemoglobin mass are correlated with critical power and maximal oxygen consumption [10, 13, 14], (2) infusion of erythrocytes or erythropoietin increases maximal oxygen consumption and performance [15–18], whereas (3) blood donation or depletion decreases oxygen transport capacity and performance [19, 20]. CD47, regulating erythrocyte removal by macrophages, influences erythrocyte number and thus oxygen transport and exercise performance [21]. Furthermore, oxygen diffusion from capillaries to muscle mitochondria depends significantly on erythrocyte flux and velocity within capillaries [22–26].

Beyond their number, erythrocytes possess specialized metabolism that actively supports their oxygen transport role. Hemoglobin oxygen affinity is fine-tuned by metabolic allosteric effectors such as 2,3-bisphosphoglycerate (2,3-BPG), a glycolytic byproduct [9, 10, 27]. Glycolytic flux is therefore a key regulator of hemoglobin’s oxygen-binding properties. In clinical and storage contexts, bioenergetic enhancers have been used to improve erythrocyte metabolism and overall oxygen kinetics [28–34]. For instance, patients with erythrocyte metabolic impairments benefit from pyruvate kinase activators, which enhance glycolytic flux and oxygen transport [31, 35].

Erythrocyte redox status is tightly linked to oxygen transport by modulating glycolysis and allosteric effectors, preserving reduced hemoglobin and maintaining cell deformability [9]. Under oxidative stress, whether owing to storage or diseases, key redox regulators (e.g., glutathione, and vitamins C and E) are depleted, impairing glycolytic flux and oxygen delivery [36–42]. Oxidative stress also modifies glycolytic enzymes directly, further reducing metabolic efficiency [43–45]. Energy and reducing equivalents from both glycolysis and the pentose phosphate pathway are essential to sustain redox status [46, 47]. Thus, erythrocyte glycolytic and redox metabolism are interconnected and regulate oxygen kinetics.

**Fig. 1:**
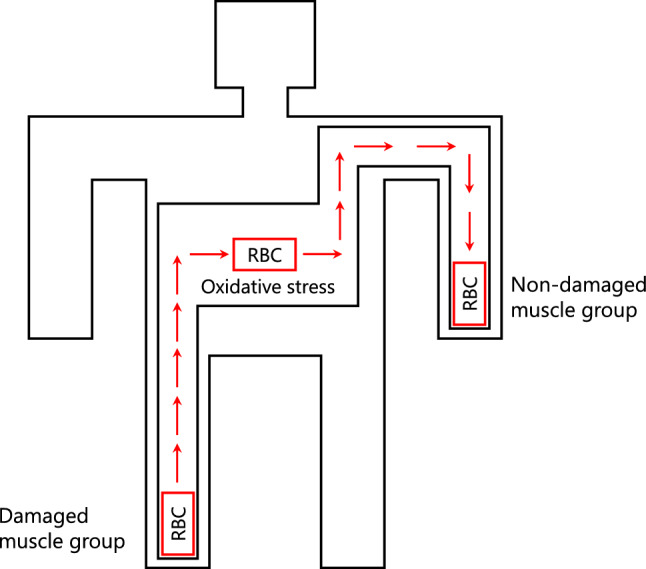
Conceptual minimalistic model of the single-leg eccentric protocol and arm exercise assessment. To investigate the systemic role of the erythrocyte metabolism, we used eccentric exercise to induce long-lasting oxidative stress in erythrocytes and evaluated the effect on muscle oxygenation and performance of the arm muscles

Despite theoretical evidence, erythrocyte metabolic contributions to oxygen delivery during exercise have not been comprehensively investigated. To address this, we used a single-leg eccentric contraction protocol known to induce long-lasting low-grade oxidative stress in erythrocytes [48–52], enabling in vivo probing of erythrocyte metabolism while isolating its potential contribution to oxygenation and performance in distant arm muscles in a randomized cross-over design (Fig. 1). Combining in vivo, ex vivo, and computational approaches, we aimed to clarify the link between erythrocyte metabolism and skeletal muscle physiology under normal and oxidative stress conditions.

## Methods

### Participants

A total of 20 (*n* = 20) active young male volunteers were enrolled in the study (mean ± SD; 23 ± 4 years, 76.4 ± 8.77 kg, 178 ± 6 cm, 24.10 ± 2.61 kg/m^2^, 14.95 ± 4.53% body fat). To our knowledge, no other study has investigated the role of the erythrocyte glycolytic flux in exercise performance. Hence, it was not possible to determine the sample size on the basis of the erythrocyte glycolytic flux. The required sample size was calculated using G*Power 3.1 [53] to obtain a power of 0.80 or higher and alpha level of 0.05 for the primary muscle damage (e.g., isometric torque, delayed onset muscle soreness) [54] and redox biomarkers (e.g., glutathione, protein carbonyls) [52]. Musculoskeletal injuries, chronic diseases, smoking, consumption of any sports/antioxidant supplements or medication, and potential inflammatory conditions comprised the exclusion criteria. Participants were instructed to abstain from any strenuous exercise during the study period. Finally, participants were asked to record their diet the day prior to the first lab visit and to follow the same diet the day before each visit. The study methods were approved by the Ethics Committee of the Department of Physical Education and Sports Science at Serres (ERC-017/2022). The study protocol was also preregistered on the Open Science Framework (https://osf.io/ub6zs). Data was collected at Aristotle University of Thessaloniki between May 2023 and August 2023. Participants provided written informed consent and all procedures conformed to the guidance presented by the Declaration of Helsinki [55].

### Study Design

To investigate the role of erythrocyte metabolism in exercise physiology, we used the single-leg eccentric contraction protocol to induce long-lasting oxidative stress in erythrocytes and evaluated the effect on the glycolytic and redox metabolism, as well as on muscle oxygenation and exercise performance, using the distant nondamaged arm muscles (Fig. 1). The single-leg model exploits the methodological and statistical advantages of the single-limb exercise model [56].

**Fig. 2:**
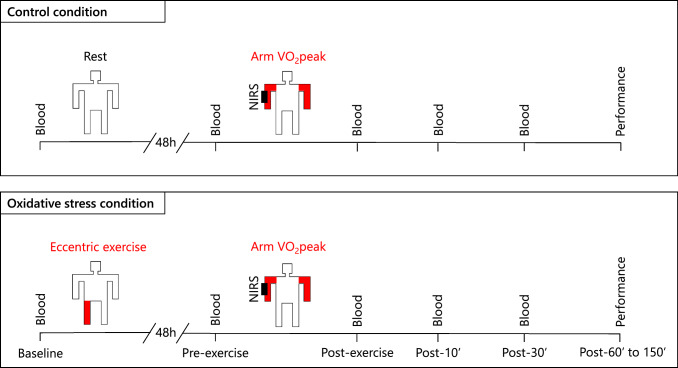
Study design. A total of 20 male participants underwent a randomized crossover experiment with two conditions: oxidative stress (eccentric contractions) and control. Oxidative stress was induced via leg eccentric contractions, and its effects on erythrocyte glycolytic and redox metabolism, arm muscle oxygenation, and arm exercise performance were evaluated

A research assistant, not involved in data collection and analysis, allocated the participants to the control (absence of oxidative stress) and oxidative stress condition, in a double-blind randomized crossover design (Fig. 2), using the Minirand package in R (Version 4.4.2; R Core Team, 2024). The participants visited the lab twice for each condition, and the two conditions were separated by at least 3 weeks to ensure adequate wash-out. During the first visit, participants in the oxidative stress condition performed a single-leg eccentric protocol (75 maximal knee extensor contractions) in the isokinetic dynamometer to induce oxidative stress. In contrast, in the control condition participants rested seated in the dynamometer for approximately 15 min. The experimental leg was randomly chosen while ensuring balanced allocation of the dominant and nondominant legs, using minimization randomization with the Minirand package. At 2 days after the first visit, in both conditions, participants performed a cardiopulmonary exercise test in the arm ergometer to evaluate the peak oxygen uptake (VO_2_peak) using the distant and nondamaged muscle group of the arm. Subsequently, VO_2_peak was also measured in the cycle ergometer, while arm and leg muscle performance were evaluated in the isokinetic dynamometer. We used near-infrared spectroscopy to estimate the erythrocyte’s capacity to transport and deliver oxygen and evaluated arm and leg muscle oxygenation, during the arm and leg cardiopulmonary tests, respectively. Vascular occlusion near-infrared spectroscopy tests were also performed in the arm and leg muscles to assess microcirculatory function. Blood samples were collected at baseline before each condition, as well as 2 days later, before and after 0-, 10- and 30-min of the arm ergometer exercise. The rate of erythrocyte lactate production was measured ex vivo at 0, 30, 60, and 90 min to calculate the glycolytic flux [57–59]. To investigate the potential mechanistic effects of hydrogen peroxide and glucose on glycolytic flux, we conducted ex vivo experiments by incubating cells with exogenous hydrogen peroxide and glucose. Finally, the collected erythrocytes were used to measure: (1) general blood count; (2) glycolytic metabolites and enzymes; (3) redox metabolites and enzymes; and (4) oxidative stress biomarkers. The collected blood was used for the evaluation of (1) complete blood count, (2) plasma hemoglobin, (3) methemoglobin; and (4) creatine kinase.

### Cardiopulmonary Testing

The arm incremental test was performed on an upper body ergometer (881E Upper Body Ergometer, Monark, Vansbro, Sweden). The leg incremental test to exhaustion was performed on a cycle ergometer (894E Peak Bike, Monark, Vansbro, Sweden) as described previously [60]. All participants were familiarized with incremental exercise testing in the arm and cycle ergometer. The familiarization session consisted of a shorter version using only the first three stages of the test. Participants cycled for 5 min at 100 W for warm-up. Subsequently, the workload increased by 50 W every 2.5 min until the heart rate reached 160 bpm, and then it increased by 25 W every 2.5 min until exhaustion. The arm incremental test consisted of a 5 min warm-up using 1% of bodyweight. After the warm-up, the workload increased by 20 W every 2.5 min until exhaustion. Both tests were terminated when three of the following four criteria were met: (1) volitional fatigue, (2) an increase in VO_2_ of less than 2 mL/kg/min despite an increase in workload, (3) a respiratory exchange ratio equal to or greater than 1.10, and (4) a heart rate within 10 bpm of the predicted maximal heart rate [HR_max_ = 208 − (0.7 × age)]. Respiratory gas volume signals were measured using a metabolic cart (Quark RMR, Cosmed, Italy), which was calibrated according to manufacturer’s recommendation before each test. The end-exercise values for the cardiopulmonary variables (i.e., VO_2_peak, VCO_2_peak_,_ RERpeak, and HRpeak) were determined from the average of the final 30 s of exercise.

### Isokinetic Dynamometry

The single-leg eccentric protocol and the isokinetic assessments of arms and legs were performed in the same isokinetic dynamometer (Humac Norm, Ronkonkoma, NY). The isokinetic dynamometer was calibrated according to the manufacturer’s instructions. Before study initiation, all participants visited the lab to familiarize themselves with the arm and cycle ergometer and isokinetic dynamometry testing protocols to avoid any learning effects. The familiarization with the isokinetic dynamometer consisted of 2 × 4 s submaximal voluntary isometric, concentric, and eccentric contractions of the elbow flexors and knee extensors. All isokinetic tests were performed from a seated position (60° hip angle with 0° representing the anatomical position of the hip joint). During the isokinetic leg protocols, the lateral femoral epicondyle was aligned with the axis of rotation of the dynamometer, and participants were coupled to the dynamometer by an ankle cuff attached proximally to the lateral malleolus. Each participant’s functional range of leg motion was set electronically between 0° of full extension and 90° of knee flexion. During the isokinetic arm protocols, the seat and the dynamometer were adjusted to ensure that the lateral humerus epicondyle aligned with the axis of rotation of the dynamometer. The shoulder was positioned at 30° flexion and slight abduction, while the arm was supported on a soft pad. The participant held the dynamometer arm with a supinated grip. The anatomical zero of the elbow joint was set at 0° full extension. Gravitational corrections were made to account for the effect of the arm and leg mass on torque measurements. Prior to the leg and arm isokinetic tests, participants performed 8 min of warm-up in the cycle and arm ergometer, respectively.

In the single-leg eccentric contraction protocol, participants completed a moderate exercise volume consisting of five sets of 15 eccentric contractions (75 contractions in total) at maximal voluntary contraction with the knee extensors of the experimental leg, at an angular velocity of 60°/s. Subjects were verbally encouraged to maximally activate their knee extensors throughout the protocol. Exactly 1 min rest intervals were utilized between sets.

Maximal isometric, concentric, and eccentric isokinetic torque were also measured to evaluate the leg and arm muscle performance. The maximal concentric and eccentric tests of knee extensors consisted of 3 × 7 maximal voluntary contractions (0° full extension and 120° of elbow flexion). The maximal concentric and eccentric tests of elbow flexors consisted of 3 × 7 maximal voluntary contractions (0° of full extension and 90° of knee flexion). The evaluation of the maximal isometric knee extensor torque at 90° and elbow flexor torque at 90° consisted of 3 × 7 s maximal voluntary contractions. Each set was separated by 1 min of rest. The highest maximal torque contraction was recorded at each test. To ensure that the subjects provided their maximal effort, the tests were repeated if the difference between the lower and the higher recorded maximal torque exceeded 10%.

### Muscle Damage Biomarkers

Pain-free range of movement (ROM) and delayed onset muscle soreness (DOMS) were used as indirect biomarkers of muscle damage, because they are considered reliable and valid indices for the evaluation of exercise-induced muscle damage [54]. ROM was performed manually while the subjects remained seated on the isokinetic dynamometer. For the leg ROM, the investigator moved the calf at a very low angular velocity from 0° knee extension to the position where the subject felt any discomfort. For the arm ROM, the investigator moved the hand at a very low angular velocity from 130° elbow flexion to the position where the subject felt any discomfort. From the same seated position, participants performed unloaded knee extension and elbow flexion movement to assess the DOMS in the leg and arm, respectively. Perceived muscle soreness was rated on a scale ranging from 1 (normal) to 10 (very sore) [61, 62].

### Near-Infrared Spectroscopy

Continuous-wave near-infrared spectroscopy (NIRS) was used to noninvasively assess muscle oxygenation during the arm and leg incremental tests, in both conditions, as previously described [63]. The NIRS device consists of three optode sources, each emitting two near-infrared lights, at 760–850 nm wavelengths, and one optical receiver aligned with the optodes. The interoptode distance between the receiver and optodes is 30, 35, and 40 mm, while the penetration depth is half the distance between the receiver and optodes, that is, 15, 17.5, and 20 mm [64, 65]. Continuous-wave NIRS cannot discriminate between chromophores, that is, hemoglobin and myoglobin, while myoglobin content within the muscle tends to remain constant during exercise [66, 67]. Therefore, the NIRS device provides microvascular changes in oxygenated hemoglobin (O_2_Hb) and deoxygenated hemoglobin (HHb) concentrations and their sum (total hemoglobin; tHb).

For the arm, the NIRS device was placed at the medial side over the midbelly of the biceps brachii. For the leg, the NIRS device was placed at the lower third of vastus lateralis muscle, approximately 12 cm above the patella and 5 cm lateral to the midline, following shaving and cleaning of the site with an alcohol swab. In both the arm and leg measurements, the NIRS device was covered in a transparent elastic wrap, fixated using adhesive tape and covered with a black sleeve, to minimize movement artifacts and block exogenous light. The same experimenter placed the device at all measurements and marked the placement each time to ensure accuracy and reproducibility. The bicep brachii and vastus lateralis muscles were selected owing to their accessibility and quality of the signals, as determined in pilot measurements. Adipose tissue thickness of vastus lateralis and biceps brachii (i.e., skinfold thickness/2) was determined using a skinfold caliper (Harpenden, John Bull, St. Albans, England). The average adipose tissue thickness over the biceps brachii (3.94 ± 1.17 mm) and right vastus lateralis muscle (5.71 ± 2.75 mm), were well below the minimum NIRS light penetration depth (i.e., 15 mm) and therefore did not influence the amplitude of the NIRS signal. Finally, NIRS was used during vascular occlusion testing to assess the microvascular function of the arm and leg [68–70]. The vascular occlusion test consisted of 5 min rest, 5 min arterial occlusion, and 3 min of recovery.

The NIRS signals were measured continuously at a frequency of 10 Hz during three distinct phases: (1) rest; (2) exercise/occlusion; and (3) recovery. Data were collected and analyzed using Oxysoft software (version 2.3.70; Artinis Medical Systems). The average of the three optode signals were used for analyses. During a 5 min rest period, prior to each exercise/occlusion protocol, baseline values were obtained from the average of the last 30 s. Owing to the different exercise duration times between participants in the arm and leg incremental tests, the total time of exercise was divided into 10% intervals [71], and the NIRS signals were averaged at 30 s bins at each time point, as described previously [72]. Recovery was measured at 60, 90, and 120 s after both the exercise and occlusion protocol.

### Metabolic and Redox Assays

Blood collection for erythrocyte glycolytic and redox metabolic measurements was performed between 08:00 and 11:00 am, following a 12 h overnight fast. Blood samples were collected at baseline before each condition, as well as 2 days later, immediately before and after the arm ergometer exercise. Blood samples were drawn from a forearm antecubital vein, collected in ethylenediaminetetraacetic acid (EDTA) tubes and centrifuged immediately at 1370 × *g* for 10 min at 4 °C and the plasma was collected in multiple aliquots. Packed erythrocytes were lysed with 1:1 (v/v) distilled water, inverted vigorously and centrifuged at 4000 × *g* for 15 min at 4 °C. All samples were stored at − 80 °C and thawed only once before analysis. A competitive immunoassay was used for the measurement of erythrocyte F_2_-isoprostanes (Cayman Chemical, Charlotte, USA). Erythrocyte protein carbonyls, glutathione, glutathione peroxidase, glutathione reductase, superoxide dismutase, and catalase were measured using spectrophotometry [73]. Erythrocyte vitamin C was measured by high performance liquid chromatography (HPLC) with an ultraviolet detector with the use of meta-phosphoric acid as a stabilizer using a modified protocol of Kandár et al. [74]. Vitamin E was measured using HPLC with an ultraviolet detector using a modified protocol of Talwar et al. [75]. The erythrocyte hexokinase and phosphofructokinase [59], glyceraldehyde 3-phosphate dehydrogenase (GAPDH), glucose-6-phosphate dehydrogenase (G6PD), 2,3-bisphosphoglycerate (2,3-BPG) [76], and methemoglobin [77] were measured spectrophotometrically. Plasma hemoglobin was assayed using a kit from BioAssays System (Hayward, CA). Creatine kinase activity was measured using a kit from Spinreact (Sant Esteve, Spain). Erythrocyte, white blood cell, platelet count, and hematocrit were measured using an automatic analyzer. Plasma pH measurement was performed in fresh whole blood using a pH-meter (HI2002-02 edge, Hanna, Athens).

### Ex Vivo Glycolytic Flux

For the measurement of the erythrocyte glycolytic flux, blood samples were collected at the first visit of each condition, as well as 2 days later, immediately before and after 0, 10, and 30 min of the arm ergometer exercise. The glycolytic flux was quantified by measuring the concentration of extracellular lactate secreted by erythrocytes ex vivo as a function of incubation time. Erythrocytes were incubated for 0, 30, 60, and 90 min. The glycolytic flux was determined by calculating the slope of the linear regression between lactate concentration and incubation time. The rate of lactate production is used as a proxy for glycolytic flux [57] because it is easier to measure and more precise compared with measuring the rate of glucose uptake [58]. For each experimental condition, glycolytic flux was measured at rest, after the arm incremental exercise, and during the exogenous challenges. The collection and handling of erythrocytes and the measurement of glycolytic flux were carried out as described previously [57]. Fresh erythrocytes were used in all measurements.

All steps of the collection and assay were carried out at room temperature except where indicated. Blood was collected from a forearm antecubital vein; 15 mL of whole blood was collected in EDTA tubes and 1 mL of whole blood was transferred in ten Eppendorf tubes. The EDTA tube (containing 5 mL of whole blood) and the Eppendorf tubes were centrifuged immediately at 500 × *g* for 10 min at 4 °C. The erythrocytes and plasma from the EDTA tubes were collected and stored at − 80 °C. Each Eppendorf tube was split into four aliquots, one for each incubation time. The erythrocyte pellet in the Eppendorf tubes was washed with the erythrocyte buffer (1:1) and inverted gently to mix the content. The erythrocyte buffer was supplemented with 10 mM of glucose. Then, it was centrifuged again at 500 × *g* for 10 min at 4 °C. This washing step was repeated two additional times. The supernatant was discarded. Erythrocyte buffer was added to reach 50% hematocrit and gently mixed by inversion. Finally, erythrocytes were incubated for 0-, 30-, 60- and 90-min at 37 °C on a shaker, and subsequently centrifuged at 500 × *g* for 5 min at 4 °C. The supernatant was collected and stored. Finally, the lactate concentration of each supernatant was measured with an assay kit (Sigma-Aldrich, Darmstadt, Germany).

### Ex Vivo Glycolytic Flux Using Exogenous Challenges

Using the nonoxidatively stressed and oxidatively stressed erythrocytes collected from the in vivo experiment, we investigated how varying glucose and hydrogen peroxide levels affect the glycolytic flux. In the glucose challenge, we incubated the erythrocytes with 3.9, 5.6, 7.2, and 10 mM of glucose and measured glycolytic flux as described in Sect. 2.8. These glucose concentrations correspond to 70, 100, 130, and 180 mg/dL, resembling resting blood glucose levels and glucose supplementation schemes. In the hydrogen peroxide challenge, we incubated the erythrocytes with 10 mM glucose concentration and 0, 5, 10, 20, and 40 μM of hydrogen peroxide. These levels were selected to approximate hydrogen peroxide concentrations in plasma under normal conditions (1–5 μM) and during inflammatory disease (≈50 μM) [78].

### Computational Analysis

#### Stoichiometric Analyses

Chemical stoichiometry refers to the computational study of the reactants and products involved in a chemical reaction. To calculate the expected increase in ATP and NADPH owing to the increase in glycolytic flux, we performed a simple stoichiometric calculation [9, 79]. We modelled three different scenarios on the basis of the glycolytic flux measured experimentally during: (1) nonstressed control condition, (2) oxidative stress condition, and (3) acute exercise. In our stoichiometric model, we assumed that the pentose phosphate pathway would operate in a mode of maximal NADPH generation efficiency, supported by reverse upper glycolysis, as described in Dick and Ralser (2015) [80]. Specifically, if six glucose-6-phosphate molecules enter the pentose phosphate pathway, five glucose-6-phosphate molecules are regenerated, and one is completely oxidized to yield 12 NADPH molecules. Of course, such energetic efficiency may not be attainable in vivo, yet even partial recycling of glucose-6-phosphate molecules would result in high concentrations of NADPH. Moreover, in all conditions, if no other sources consume NADH, glycolysis results in a zero sum of NADH, since two molecules are produced and two molecules are consumed in this pathway.

#### Oxygen Dissociation Curve Analyses

We have previously created a mathematical model calculating the effect of 2,3-BPG, pH, pCO_2_, and temperature on the hemoglobin oxygen dissociation curve [9]. A custom R package was created on the basis of the mathematical equations from Dash and Bassingthwaighte [81, 82]. We used these equations to calculate the effect of 2,3-BPG and blood pH on the oxygen dissociation curve and p_50_ (i.e., partial pressure of oxygen when hemoglobin is 50% saturated with oxygen), during pre- and post-exercise in the control and oxidative stress conditions. Blood pH, 2,3-BPG, hematocrit, and hemoglobin concentrations were measured in the present study. Since we did not measure erythrocyte temperature, we used values of arterial blood temperature at rest (37 °C) and during exercise (38.5 °C), using a similar exercise model from the literature [10], and assumed that they would not be different between the two conditions. Finally, the normal values of partial pressure of oxygen (100 mmHg) and carbon dioxide (40 mmHg) were used. The data and code are available on the manuscript’s GitHub repository.

### Statistics

The normality of distribution was tested using the Shapiro–Wilk test. A two-way repeated measures analysis of variance (ANOVA) [condition (control, oxidative stress) × time (baseline, pre-exercise, and at 0-, 10-, and 30-min postexercise)] was used to evaluate the effect of oxidative stress on the erythrocyte glycolytic flux. A two-way repeated measures ANOVA [condition (control, oxidative stress) × time (baseline, pre- and postexercise)] was used to evaluate the effect of oxidative stress on erythrocyte glycolytic and redox molecules (e.g., 2,3-BPG, glutathione), enzymes (e.g., hexokinase, catalase), and blood measurements. For the near-infrared spectroscopy signals a two-way repeated measures ANOVA was performed [condition (control, oxidative stress) x exercise duration (baseline, 10–100% of exercise duration, and recovery)]. Finally, a two-way repeated measures ANOVA was performed to evaluate the effect of glucose [condition (control, oxidative stress) × dose (3.9, 5.6, 7.2, and 10 mM)] and hydrogen peroxide [condition (control, oxidative stress) × dose (0, 5, 10, 20, and 40 μM)] on glycolytic flux. When a significant interaction was found, post-hoc pairwise comparisons were performed using the Sidak test. When sphericity was violated, the Greenhouse–Geisser correction was applied if the estimates of sphericity were greater than 0.75, otherwise the Huynh–Feldt correction was preferred. Paired-sample *t*-tests were performed to compare the effect of oxidative stress on arm and leg ergometer cardiopulmonary outcomes. If the assumption of normality was violated, Wilcoxon signed rank tests were performed. The partial eta squared (η2_p_), Hedge’s *g* correction (g), and rank-biserial correlation (rb) effect sizes were calculated for the ANOVA, paired *t*-tests, and Wilcoxon signed-rank tests, respectively. Data in the figures are presented as mean and 95% confidence intervals. Statistical significance was set at *α* = 0.05. Data processing and statistical analyses were performed using R (Version 4.4.2; R Core Team, 2023), RStudio (Version 2024.09.1 + 394; RStudio Team, 2024), and IBM SPSS Statistics (Version 29; IBM, SPSS Inc.). ANOVA tests and post-hoc comparisons were performed using the afex, multcomp and emmeans packages. Effect sizes were calculated as described in Jane et al. [83] and were confirmed using dedicated R packages (effsize, effectsize, MOTE, TOSTER, rstatix). Figures were created using the tidyverse, ggbeeswarm, and patchwork packages.

## Results

The complete statistical analysis is presented in Supplementary files 1 and 2.

### Muscle Damage

#### Leg and Arm ROM and DOMS

**Fig. 3:**
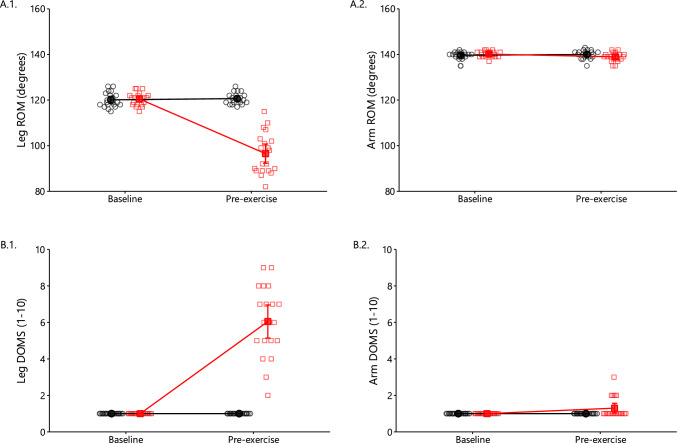
Leg and arm pain-free range of motion (**A**) and delayed onset muscle soreness (**B**) in the control (black circles) and oxidative stress condition (red squares) at baseline and pre-exercise two days later (mean ± 95% CI). Significant main effects of time, condition, and interaction were found on leg range of motion (ROM) and delayed onset muscle stiffness (DOMS; all *p* < 0.001)

A significant main effect of time, condition and interaction were found on leg ROM and DOMS (all *p* < 0.001; Fig. 3A.1 and 3B.1). At 2 days after the eccentric protocol, leg ROM decreased by − 20% and DOMS increased by + 505% compared with baseline. In general, the eccentric exercise in legs did not induce significant effects on arm ROM and DOMS.

#### Hemolysis and Creatine Kinase

**Fig. 4:**
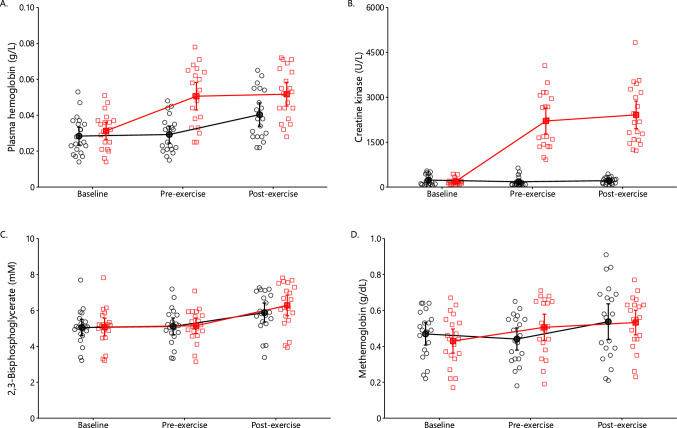
Plasma hemoglobin (**A**), plasma creatine kinase (**B**), erythrocyte 2,3-bisphosphoglycerate (**C**), and erythrocyte methemoglobin (**D**) in the control (black circles) and oxidative stress condition (red squares) at baseline as well as 2 days later at pre-exercise and postexercise (mean ± 95% CI). Significant main effects of condition (*p* = 0.004, < 0.001), time (both *p* < 0.001), and interaction (both *p* < 0.001) were observed for plasma hemoglobin (**A**) and creatine kinase (**B**). There was a significant main effect of time (*p* < 0.001) on both 2,3-BPG and methemoglobin concentrations (**D**)

A significant main effect of condition (*p* = 0.004 and *p* < 0.001, respectively), time (both *p* < 0.001), and interaction (both *p* < 0.001) were observed on plasma hemoglobin and creatine kinase (Fig. 4A and B). At 2 days after the leg eccentric protocol, plasma hemoglobin and creatine kinase levels were increased by + 62% and + 1083% and remained increased after the arm exercise by + 65% and + 1192%, compared with baseline.

### Glycolysis

#### Glycolytic Flux

**Fig. 5:**
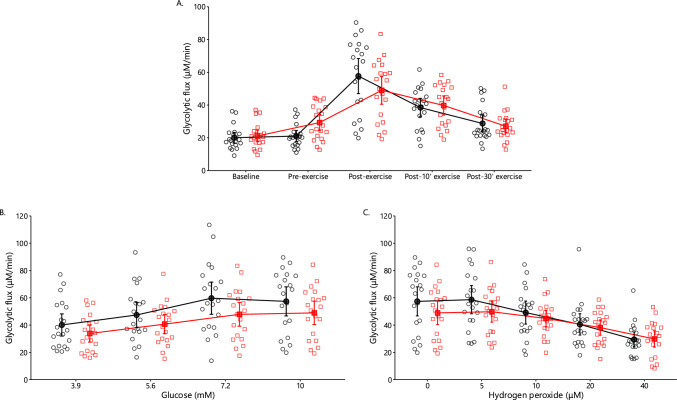
Erythrocyte glycolytic flux (**A**) in the control (black circles) and oxidative stress conditions (red squares) at baseline as well as 2 days later at pre-exercise and 0-, 15- and 30-min postexercise (mean ± 95% CI). Glycolytic flux was measured ex vivo as the rate of lactate production. Glycolytic flux during glucose (**B**) and hydrogen peroxide (**C**) ex vivo challenge in the control (black circles) and oxidative stress conditions (red squares; mean ± 95% CI). Glycolytic flux (**A**) showed a significant main effect of time (*p* < 0.001) and interaction (*p* = 0.013). Significant main effects of condition (*p* = 0.011, 0.032) and time (*p* = 0.001, *p* < 0.001) were observed for glucose (**B**) and hydrogen peroxide challenge (**C**), respectively

A significant main effect of time (*p* < 0.001) and interaction (*p* = 0.013) on glycolytic flux were observed (Fig. 5A). Leg eccentric exercise increased glycolytic flux by + 39%. Immediately after arm exercise, glycolytic flux increased by + 174% in the control and by + 67% in the oxidative stress conditions. In general, glycolytic flux returned to baseline values 30 min after arm exercise.

#### Glycolytic Flux Under Glucose and Hydrogen Peroxide Challenges

On the glucose challenge, there was a significant main effect of condition (*p* = 0.011) and time (*p* = 0.001) on glycolytic flux (Fig. 5B). Glycolytic flux was on average lower by − 16% in the oxidative stress compared with the control condition. Glycolytic flux increased by + 41% in the oxidative stress and + 49% in the control condition at 7.2 mM. On the hydrogen peroxide challenge, there was a significant main effect of condition (*p* = 0.032) and time (*p* < 0.001) on glycolytic flux (Fig. 5C). The addition of hydrogen peroxide decreased glycolytic flux in both conditions.

#### Hexokinase, Phosphofructokinase, GAPDH, and G6PD

**Fig. 6:**
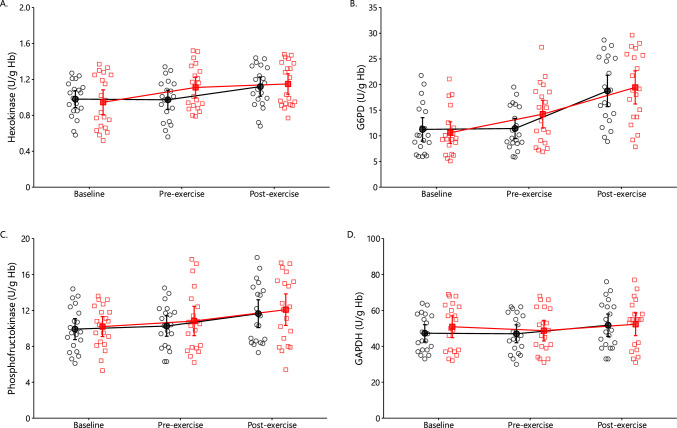
Erythrocyte hexokinase (**A**), glucose-6-phosphate dehydrogenase (G6PD; **B**), phosphofructokinase (**C**) and glyceraldehyde 3-phosphate dehydrogenase (GAPDH; **D**) activity in control (black circles) and oxidative stress conditions (red squares) at baseline as well as 2days later at pre-exercise and postexercise (mean ± 95% CI). There was a significant interaction and main effects of time on both hexokinase (*p* = 0.002; *p* < 0.001; **A**) and G6PD activity (*p* = 0.033; *p* < 0.001; **B**), respectively

There was a significant condition by time interaction (*p* = 0.002) and a main effect of time (*p* < 0.001) on hexokinase activity (Fig. 6A). At 2 days after the leg eccentric protocol, hexokinase activity increased by + 17%. Arm exercise increased hexokinase activity by + 15% in the control condition. On G6PD activity, the results revealed a significant main effect of time (*p* < 0.001) and interaction (*p* = 0.033) (Fig. 6B). Leg eccentric exercise increased G6PD activity by + 13%. Immediately after arm exercise, G6PD activity increased by + 64% in the control and by + 37% in the oxidative stress conditions. In general, phosphofructokinase and GAPDH activity did not change after leg eccentric exercise or arm exercise (Fig. 6C, D).

#### 2,3-BPG and Methemoglobin

There was a significant main effect of time (*p* < 0.001) on 2,3-BPG concentration (Fig. 4C). Arm exercise increased 2,3-BPG concentration by + 14% in the control condition and by + 22% in the oxidative stress condition. There was a significant main effect of time (*p* < 0.001) on methemoglobin concentration (Fig. 4D). In general, minimal effects were observed on methemoglobin concentration after leg eccentric or arm exercise.

### Redox Metabolism

#### F_2_-Isoprostanes, Protein Carbonyls, and Glutathione

**Fig. 7:**
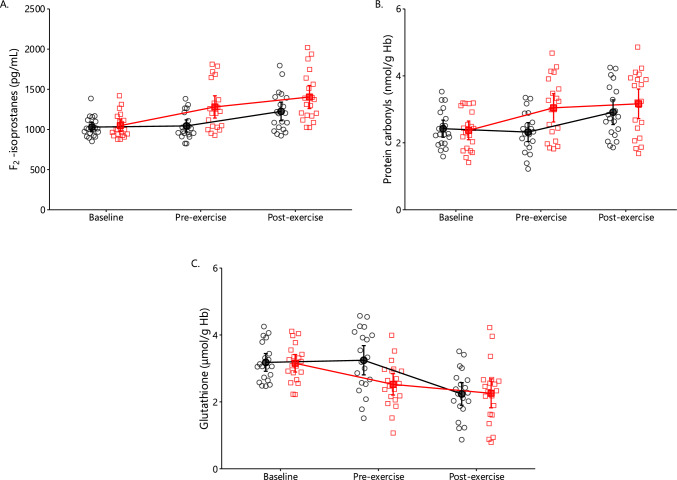
Erythrocyte F_2_-isoprostanes (**A**), protein carbonyls (**B**), and glutathione (**C**) concentration in the control (black circles) and oxidative stress conditions (red squares) at baseline as well as 2 days later at pre-exercise and postexercise (mean ± 95% CI). Significant main effects of time (all *p* < 0.001) and interactions were observed for F_2_-isoprostanes (*p* = 0.026; **A**), protein carbonyls (*p* = 0.005; **B**), and glutathione (*p* < 0.001; **C**). A main effect of condition was also found for F_2_-isoprostanes (*p* = 0.007)

A significant main effect of condition (*p* = 0.007), time (*p* < 0.001), and interaction (*p* = 0.026) was observed in F_2_-isoprostanes concentration (Fig. 7A). Leg eccentric exercise increased F_2_-isoprostanes concentration by + 22%. Arm exercise increased F_2_-isoprostanes concentration by + 17% in the control and by + 10% in the oxidative stress condition. A significant main effect of time (*p* < 0.001) and interaction (*p* = 0.005) were observed on the protein carbonyls concentration within erythrocytes (Fig. 7B). Leg eccentric exercise increased protein carbonyls concentration by + 28%. Arm exercise increased protein carbonyls concentration by + 26% in the control and by + 4% in the oxidative stress conditions. A significant main effect of time (*p* < 0.001) and interaction (*p* < 0.001) was found in the concentration of glutathione concentration in erythrocytes (Fig. 7C). Leg eccentric exercise decreased glutathione concentration by − 20%. Arm exercise decreased glutathione concentration by − 32% in the control and by − 11% in the oxidative stress conditions.

#### Superoxide Dismutase, Catalase, Glutathione Peroxidase, and Glutathione Reductase

**Fig. 8:**
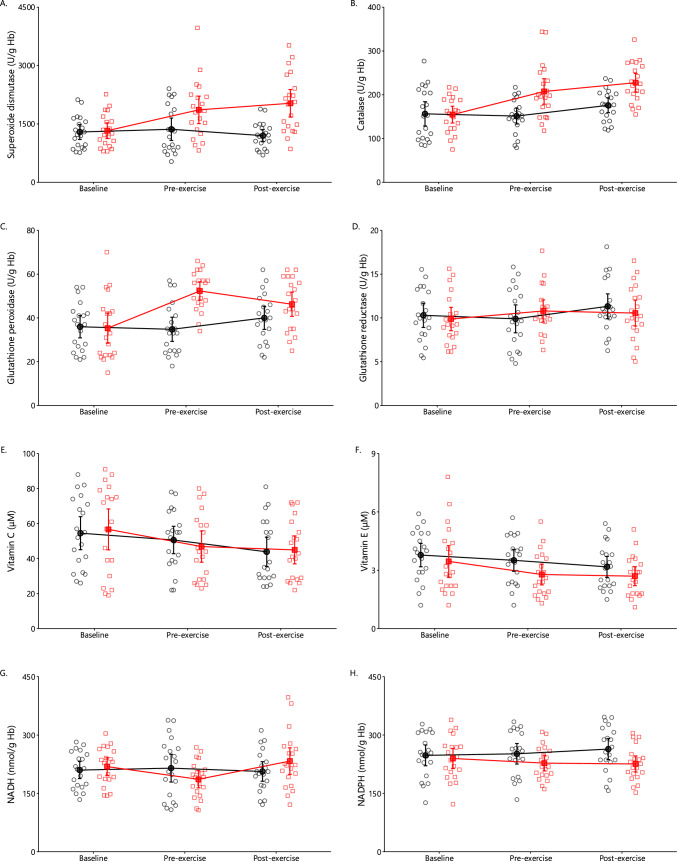
Erythrocyte superoxide dismutase (**A**), catalase (**B**), glutathione peroxidase (**C**), glutathione reductase (**D**), vitamin C (**E**), vitamin E (**F**), NADH (**G**), and NADPH (**H**) levels in the control (black circles) and oxidative stress conditions (red squares) at baseline as well as 2 days later at pre-exercise and postexercise (mean ± 95% CI). Significant main effects of time (*p* < 0.01), condition (*p* < 0.001), and condition × time interaction (*p* < 0.001) were observed for superoxide dismutase, catalase, and glutathione peroxidase activity. A significant main effect of time was found for vitamins C and E (both *p* < 0.001), while significant interactions were observed for NADH (*p* = 0.022) and NADPH (*p* = 0.039)

A significant main effect of time (*p* < 0.01), condition (*p* < 0.001), and a condition by time interaction (*p* < 0.001) were detected on superoxide dismutase, catalase, and glutathione peroxidase activity (Fig. 8A–C). Leg eccentric exercise increased the activity of superoxide dismutase by + 40%, catalase by + 34%, and glutathione peroxidase by + 48%. Arm exercise did not induce any further changes in the activity of the three enzymes. In general, glutathione reductase activity did not change either by the leg eccentric or arm exercise (Fig. 8D).

#### Vitamin C, Vitamin E, NADH, and NADPH

Regarding the nonenzymatic redox molecules, no significant interactions appeared for vitamin C and vitamin E concentration. A significant main effect of time appeared for both vitamins (*p* < 0.001), indicating a decrease in the control and oxidative stress condition (Fig. 8E and F).

Regarding NADH and NADPH, there was no significant main effect of condition or time (Fig. 8G and H). However, a significant interaction was observed in both NADH (*p* = 0.022) and NADPH (*p* = 0.039). Compared with baseline, NADH was significantly decreased by − 15%, 2 days after the oxidative stress condition (*p* < 0.001). Post-hoc tests did not show any differences in NADPH concentration.

### Exercise Near-Infrared Spectroscopy

#### Arm Oxygenation

**Fig. 9:**
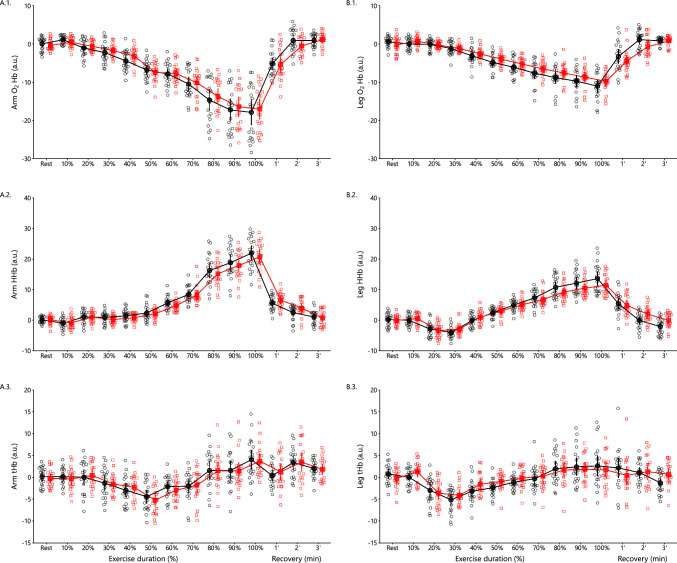
Arm muscle oxygenated (**A1**), deoxygenated (**A2**), total hemoglobin levels (**A3**), leg muscle oxygenated (**B1**) deoxygenated (**B2**), and total hemoglobin levels (**B3**) at rest, during the arm, and leg incremental test, respectively, as well as 1-, 2- and 3-min postexercise (mean ± 95% CI). Control and oxidative stress conditions are represented in black circles and red squares, respectively. Significant main effects of time (all *p* < 0.001) were observed for O₂Hb, HHb, and tHb levels in both the arm (**A1**–**A3**) and leg (**B1**–**B3**) during the incremental tests. Significant interactions were found for arm HHb (*p* = 0.036), for leg O₂Hb and HHb (both *p* < 0.001), and tHb (*p* = 0.012)

During the arm incremental test, a significant main effect of time (*p* < 0.001) was observed on arm O_2_Hb levels (Fig. 9A1). There was a significant main effect of time (*p* < 0.001) and interaction (*p* = 0.036) on HHb levels (Fig. 9A2). In both conditions, arm HHb levels were progressively increased until exhaustion and returned to baseline levels after 3 min of recovery. On tHb levels, there was a significant main effect of time (*p* < 0.001) (Fig. 9A3).

#### Leg Oxygenation

During the cycling incremental test, a significant main effect of time (*p* < 0.001) and interaction (*p* < 0.001) were observed (Fig. 9B1) on leg O_2_Hb levels. Leg O_2_Hb levels were significantly decreased from baseline until exercise exhaustion and returned to baseline levels after a 3 min recovery. After 2 min recovery, O_2_Hb reached higher levels in the control compared with the oxidative stress condition. Similarly, a significant main effect of time (*p* < 0.001) and interaction (*p* < 0.001) were found on HHb levels (Fig. 9B2). In both conditions, leg HHb levels decreased during exercise onset, then continued to increase until exhaustion and returned to baseline levels after 3 min of recovery. Finally, there was a significant main effect of time (*p* < 0.001) and interaction (*p* = 0.012) on tHb levels (Fig. 9B3). In both conditions, at exercise initiation tHb levels were first decreased and then increased progressively until exhaustion and returned to baseline levels after 2 min of recovery.

### Vascular Occlusion Near-Infrared Spectroscopy

#### Arm Occlusion

Regarding the arm vascular occlusion tests, the main effect of time (*p* < 0.001) on O_2_Hb was observed (Supplementary Fig. S1A). There was a main effect of time (*p* < 0.001) and interaction (*p* < 0.001) on HHb levels (Supplementary Fig. S1B). In both conditions, HHb levels were significantly decreased during the 5 min occlusion and recovered immediately upon reperfusion. HHb levels at 4 min and 5 min of occlusion were lower in the oxidative stress compared with the control condition. A significant main effect of time (*p* = 0.020) was observed on tHb levels (Supplementary Fig. S1C).

#### Leg Occlusion

In the leg occlusion tests, a significant main effect of condition, time, and interaction was observed on O_2_Hb levels (all *p* < 0.001) (Supplementary Fig. S2A). In both conditions, O_2_Hb levels were decreased through the 5 min occlusion and reoxygenated to baseline levels at recovery. At 2 min and 3 min of occlusion, O_2_Hb levels were lower in the control compared with oxidative stress condition but reached higher levels at 1 min of recovery. On HHb levels, there was a significant main effect of condition, time, and interaction (all *p* < 0.001) (Supplementary Fig. S2B). In both conditions, HHb levels increased throughout the occlusion and returned to baseline levels at recovery. HHb levels in the oxidative stress condition were lower compared with the control condition after 3, 4 and 5 min of occlusion. Finally, a main effect of time and interaction were detected on tHb levels (both *p* < 0.001) (Supplementary Fig. S2C). After 5 min occlusion, tHb levels were higher in the control compared with the oxidative stress condition.

### Isokinetic Dynamometry Performance

**Fig. 10:**
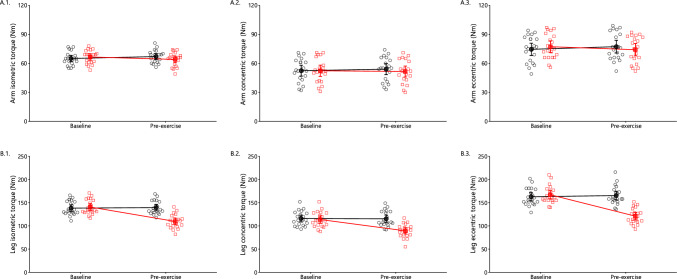
Isometric, concentric, and eccentric peak torque of the arm (**A**) and leg (**B**) in the control (black circles) and oxidative stress condition (red squares) at baseline and pre-exercise 2 days later (mean ± 95% CI). In arm dynamometry (**A**), significant interactions were observed for isometric (*p* = 0.006) and eccentric (*p* = 0.004) peak torque. For the leg (**B**), significant main effects of condition and time (all *p* < 0.001), along with interactions (all *p* < 0.001), were found for isometric, concentric, and eccentric peak torque

Regarding the arm isokinetic dynamometry (Fig. 10A), there was a significant interaction on the arm isometric (*p* = 0.006) and eccentric peak torque (*p* = 0.004). Post-hoc did not reveal any significant differences. Regarding the leg isometric, concentric, and eccentric peak torque (Fig. 10B), there was a significant main effect of condition (all *p* < 0.001) and time (all *p* < 0.001), as well as interaction (all *p* < 0.001). In the oxidative stress condition, 2 days after the eccentric protocol, the peak torque was significantly decreased compared with baseline in the isometric, concentric, and eccentric protocols. No changes were observed in the control condition.

### Peak Oxygen Uptake

**Fig. 11:**
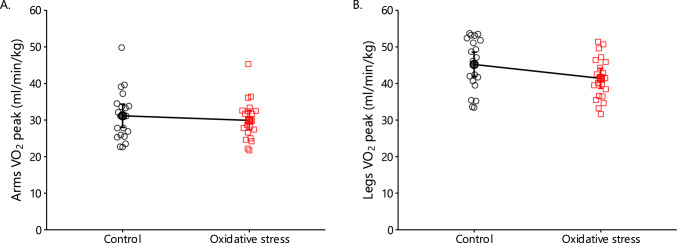
VO_2_peak during the arm (**A**) and leg (**B**) incremental test in the control (black circles) and oxidative stress condition (red squares; mean ± 95% CI). VO₂peak was reduced under oxidative stress compared with control in both the arm (*p* = 0.024) and leg tests (*p* = 0.001; Wilcoxon signed-rank)

In the oxidative stress condition, the arm VO_2_peak was lower by − 4% compared with the control condition (*p* = 0.024), even though the oxidative stress was induced by performing exercise with the leg (Fig. 11A). A Wilcoxon signed-rank test was performed on leg VO_2_peak, which was found to be lower by − 8% in the oxidative stress condition compared with the control (*p* = 0.001), probably owing to the muscle damage induced in the leg (Fig. 11B). In the arm incremental test, peak RER did not differ between the two conditions, but it was lower in the oxidative stress compared with the control condition in the cycling incremental test (*p* = 0.004). Peak heart rate values during the arm and leg incremental tests were not different between the two conditions.

### Computational Analysis

#### Increased Glycolytic Flux Leads to Higher Levels of ATP and NADPH

**Fig. 12:**
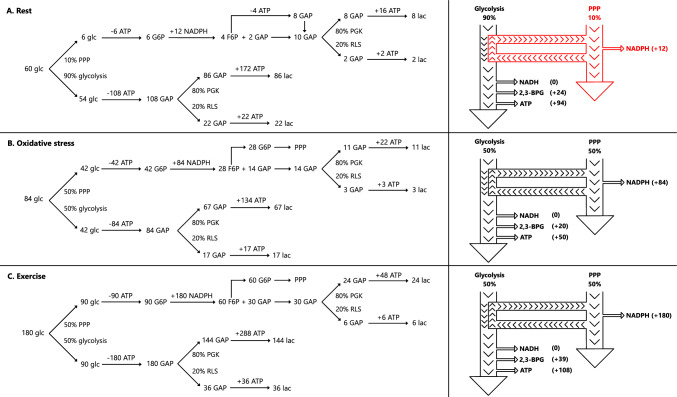
A stoichiometric analysis showing the effect of oxidative stress and exercise on the erythrocyte ATP, NADPH, and 2,3-BPG molecules. In the left panel, a stoichiometric analysis of the erythrocyte glycolysis and pentose phosphate pathways is presented, during control nonstress condition (**A**), oxidative stress condition (**B**), and during acute exercise (**C**). In the right panel, the direction of each pathway is displayed with arrows and the flux pathway weightings are presented in black, when up to 90% of glucose is utilized by the pathway, and in red when 10% of glucose is utilized by the pathway. The stoichiometric model is built on Puckeridge and colleagues as described in Chatzinikolaou et al. [9]. **A** Control: 90% of glucose is utilized by glycolysis and 10% by the pentose phosphate pathway. The results show that there is a net gain of 24 2,3-BPG, 94 ATP and 12 NADPH molecules during nonstressed resting conditions. **B** Oxidative stress: glycolytic flux was increased by 1.4-fold owing to oxidative stress per se, increasing the initial number of glucose molecules from 60 to 84 molecules. In this condition, approximately 50% of glucose is utilized by glycolysis and 50% by the pentose phosphate pathway. The results show there is a net gain of 20 2,3-BPG, 50 ATP, and 84 NADPH molecules during oxidative stress. **C** Exercise: glycolytic flux was increased by threefold owing to oxidative stress per se, increasing the initial number of glucose molecules from 60 to 180 molecules. In this condition, approximately 50% of glucose is utilized by glycolysis and 50% by the pentose phosphate pathway. The results show that there is a net gain of 39 2,3-BPG, 108 ATP, and 180 NADPH molecules during acute exercise. Abbreviations: 2,3-BPG: 2,3-bisphosphoclycerate; F6P: fructose-6-phosphate; G6P: glucose-6-phosphate; GAP: glyceraldehyde-3-phosphate; glc: glucose; lac: lactate; PPP: pentose phosphate pathway; PGK: phosphoglycerate kinase; RLS: Rapoport–Luebering shunt

During nonstressed conditions, 90% of glucose is directed to glycolysis, from which 20% of glucose skips an ATP-generating step to produce 2,3-BPG, while the remaining 10% is used by the pentose phosphate pathway to generate NADPH [79] (Fig. 12A). In this condition, there is a net gain of 24 2,3-BPG, 94 ATP, and 12 NADPH molecules per 60 glucose molecules. Oxidative stress has been reported to activate the pentose phosphate pathway by increasing glucose uptake and rerouting to this pathway to maintain redox homeostasis. In our study, glycolytic flux was 1.4-fold higher in the oxidative stress compared with the control conditions. The increased glycolytic flux resulted in a net gain of 20 2,3-BPG, 50 ATP, and 84 NADPH molecules per 84 glucose molecules (Fig. 12B). Compared with the nonstressed condition, ATP production is theoretically lower (94 versus 50 ATP), but NADPH is higher, which is necessary to support redox homeostasis. Acute exercise led to a threefold increase in glycolytic flux, resulting in a net gain of 39 2,3-BPG, 108 ATP, and 180 NADPH molecules per 180 glucose molecules (Fig. 12C). On a physiological note, the metabolic demands of erythrocytes are increased during exercise to match the metabolic demands of active tissues, such as the skeletal muscles.

#### Comparison of Erythrocyte and Muscle Tissue Energetics

Erythrocyte glycolytic flux increased 1.4-fold under oxidative stress (from 21.1 to 29.3 μM/min) and threefold following maximal arm exercise (from 21.0 to 57.7 μM/min). In contrast, skeletal muscle glycolytic flux, reported at 10 ± 13 μM/min at rest, can rise to 1340 ± 810 μM/min during a 10-min hand ergometer test at 10 kpm/min [84]. These findings align with established data showing that skeletal muscle glycolytic flux can increase up to 100-fold during maximal exercise compared with resting conditions [85]. At rest, glycolytic flux in erythrocytes and skeletal muscle appears comparable. However, during maximal exercise, skeletal muscle exhibits a 100-fold increase, whereas erythrocytes show only a threefold rise.

This disparity extends to ATP turnover rates. In skeletal muscle, ATP turnover is 24–42 mM/h at rest, increasing to 13,200 mM/h during electrically stimulated contractions and 18,000 mM/h during maximal cycling exercise [86]. In erythrocytes, resting ATP turnover is approximately 1.50 mM/h [87]. Assuming glucose utilization for ATP synthesis mirrors the observed threefold glycolytic flux increase, erythrocyte ATP turnover would reach 4.5 mM/h after exercise. This equates to just ~ 0.025% of the muscle’s ATP turnover during maximal exercise. These insights highlight the erythrocyte’s metabolic efficiency compared with skeletal muscle, underscoring its role as a metabolically “cheap” cell.

#### Changes in the Hemoglobin Allosteric Effectors Can Partly Explain the Decrease in Arm Muscle Oxygenation and VO_2_peak Changes

In blood pH, a significant main effect of time was observed (*p* < 0.001). There was not a significant interaction or condition (Supplementary Fig. S3).

We used the mathematical equations by Dash & Bassingwaighte to calculate the p_50_ for each condition and at each time point. Next, we performed a two-way repeated measures ANOVA on the p_50_ data. There was a significant main effect of time (*p* < 0.001). There was not a significant interaction or main effect of condition. Compared with pre-exercise, p_50_ was significantly higher in response to exercise in both the control and oxidative stress conditions.

**Fig. 13:**
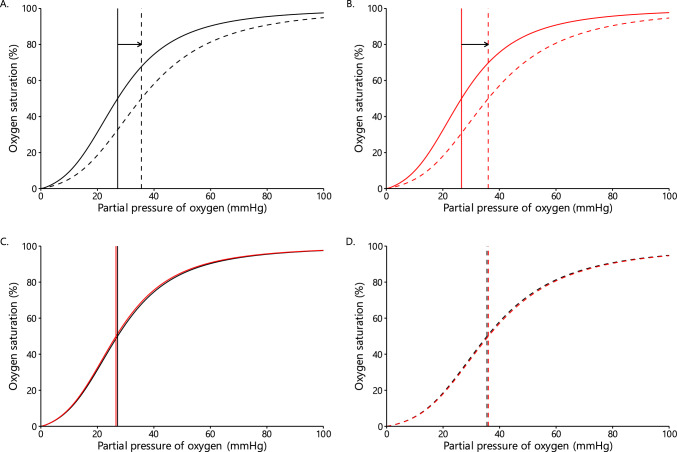
Hemoglobin oxygen dissociation curve and p_50_ (vertical lines) at pre- and postexercise in the control (**A**) and oxidative stress (**B**) condition, as well as pre- versus postexercise in the control (**C**) and oxidative stress (**D**) condition. A shift of the oxygen dissociation curve to the right facilitates greater oxygen release from hemoglobin. Pre- and postexercise data are displayed with solid and dashed lines, respectively, while the control and oxidative stress conditions are displayed in black and red color, respectively. A significant main effect of time was observed (*p* < 0.001)

Based on our calculations, p_50_ values were increased from pre-exercise to postexercise in both conditions (from 27.15 to 35.54 mmHg and from 26.59 to 36.05 mmHg in the control and oxidative stress conditions, respectively). The oxygen dissociation curve was slightly right-shifted in the oxidative stress compared with the control condition (Fig. 13C). The right-shift of the oxygen dissociation curve indicates decreased hemoglobin oxygen affinity in blood, that is, lower oxygen carrying capacity. Therefore, our mathematical calculations show that the oxidative stress conditions could have resulted in slightly lower oxygen carrying capacity, compared with the control condition, which may partly explain the lower oxygenation and VO_2_peak. Of course, other factors that we did not measure may have also influenced the hemoglobin binding properties and/or the erythrocyte deformability.

#### Minimal Effects of Oxidative Stress and Exercise on Hemolysis and Methemoglobin Levels

Exercise-induced hemolysis resulted in 149.98 g/L plasma hemoglobin (150 g/L minus 0.02 g/L), with negligible effects on oxygen carrying capacity. Methemoglobin increased from 0.43 to 0.51 (+ 0.08) owing to oxidative stress and reached 0.54 (+ 0.11) and 0.53 (+ 0.10) in the control and oxidative stress conditions, respectively. In terms of total hemoglobin, methemoglobin represented 2.9% of total hemoglobin at baseline and 3.5% of total hemoglobin after exercise. Therefore, in the best-case scenario, hemolysis and the increase in methemoglobin levels can only negligibly explain the decrease in muscle oxygenation and performance.

## Discussion

### Basic Findings

Despite extensive research, the mechanisms underlying exercise fatigue remain unclear. This is not surprising, as fatigue involves numerous biological processes across multiple tissues and organs [88]. While the responses of most organs to exercise have been studied in depth, research on erythrocytes has mainly focused on quantitative markers such as hematocrit and hemoglobin, often overlooking metabolic and systems-level changes. Even comprehensive reviews and integrative original studies on exercise physiology understandably omit erythrocyte biology [89–91], despite widespread agreement among exercise physiologists that erythrocyte count is a key determinant of endurance performance [13, 14, 19, 21, 24]. To our knowledge, this is the most comprehensive exercise study to date specifically designed to investigate possible causal links in erythrocyte biology. Our findings show that erythrocyte metabolism (1) responds dynamically to exercise, (2) becomes dysregulated under oxidative stress, and (3) may partly influence muscle oxygenation and performance.

### Eccentric Contractions Induced Oxidative Stress in the Erythrocyte at Rest

Using the single-leg eccentric model, we induced oxidative stress in erythrocytes lasting at least 2 days. This was evidenced by a 22.7% increase in F_2_-isoprostanes (lipid peroxidation marker), a 31.2% increase in protein carbonyls (protein oxidation marker), and a 22.3% decrease in glutathione (a key redox regulator) at the pre-exercise time point (2 days posteccentric model). This oxidative stress was paralleled by metabolic changes in erythrocytes, including a 39.0% increase in glycolytic flux, a 14.1% increase in hexokinase activity (a key regulator of glycolysis), and a 24.5% increase in G6PD activity (a key regulator of the pentose phosphate pathway) at the same time point.

Notably, lipid peroxidation and protein oxidation levels were higher during arm exercise under oxidative stress conditions compared with the control. We also observed reduced levels of vitamin E and glutathione, both essential for regulating hydrogen peroxide and maintaining lipid membrane integrity [50]. Erythrocyte redox status is critical for preserving deformability; with a normal diameter of 8 μm, erythrocytes must compress to less than 4–6 μm in muscle capillaries [9, 92] and 2–3 μm in splenic slits [93]. Thus, prolonged oxidative stress may have impaired deformability by damaging the membrane and cytoskeleton.

### Erythrocyte Energy and Redox Metabolism During Arm Exercise

Our study is the first to explore how erythrocyte glycolysis responds to exercise. In what is likely the most important finding of this paper, we observed that erythrocyte glycolytic flux increased by 175% after acute arm exercise in the control condition and by 67% in the oxidative stress condition, returning to baseline after 30 min of recovery (Fig. 5A). This finding reflects the dynamic nature of erythrocyte metabolism, which rises to meet increased metabolic demands during exercise to maintain energy charge, similar to other cells in the human body [94]. The reduced response of glycolytic flux under oxidative stress (67% versus 175%) suggests that oxidative stress impairs glycolytic control in erythrocytes.

Interestingly, the higher increase in glycolytic flux under control nonoxidative stress condition cannot be fully explained by the relatively stable activity of the three key glycolytic enzymes (hexokinase, phosphofructokinase, and GAPDH) or the levels of reducing cofactors (NADH and NADPH) measured in this study. This indicates that merely measuring the activity of rate-limiting enzymes or the concentration of redox cofactors does not provide a comprehensive understanding of glycolytic flux, as it is influenced by multiple control points, feedback/feedforward mechanisms, and integration with other metabolic pathways [95, 96].

Our ex vivo experiments provide indirect mechanistic support for the observed increase in glycolytic flux under nonoxidative stress conditions. The normal concentration of hydrogen peroxide in blood plasma is 1–5 μM [78]. Incrementally adding hydrogen peroxide to the medium up to 40 μM decreased glycolytic flux by approximately 50% in both conditions, confirming that oxidative stress impairs glycolysis. Prolonged oxidative stress from eccentric contractions likely oxidized glucose transporter 1, thereby reducing glucose uptake [39]. Therefore, oxidative stress may have triggered metabolic reprogramming, redirecting glucose flux to the pentose phosphate pathway.

In our stoichiometric analysis, we simulated the exercise-induced increase in glycolytic flux under oxidative stress and control conditions (Fig. 12). Postexercise, the glycolysis flux under oxidative stress reached 85.1% of the maximal flux attained in the control condition (49.0 versus 57.7 μM/min). This indicates that, at its most critical bioenergetic point, the erythrocyte produced 14.9% less ATP, NADPH, and 2,3-BPG than its theoretical maximum. While ATP was not measured, NADPH concentration was found to be 14.5% lower in the oxidative stress condition postexercise, and 2,3-BPG levels were only numerically 8.1% lower compared with the control condition.

This bioenergetic crisis in erythrocytes under oxidative stress indicates that their deformability might be affected, as changes in cell shape require energy. Membrane proteins and ion pumps, vital for adapting to osmotic and mechanical stress, consume about 50% of total cellular ATP [97]. During high-intensity exercise, erythrocytes complete more lung-to-lung circulations and undergo more deformations per cycle, with capillary flow increasing from ~ 12 cells/s at rest to ~ 40 cells/s [98, 99]. This raises bioenergetic demands, requiring a rise in glycolytic flux, similar to muscle cells, to sustain ATP production. If erythrocyte glycolytic flux fails to increase, deformability is impaired, hindering their movement through microcirculation. This is critical in vivo, as even 1–5% of erythrocytes with poor deformability can disrupt blood flow [100], reducing oxygen delivery to muscles and contributing to lower muscle oxygenation and VO₂peak. Thus, oxidative stress may impair deformability directly via membrane effects and/or indirectly by limiting glycolytic ATP production needed by mechanosensitive channels.

### Effect of Oxidative Stress and Exercise on Hemoglobin Oxygen Affinity

Using the mathematical model developed by Dash and Bassingthwaighte (2016), we explored the impact of 2,3-BPG and blood pH on the oxygen dissociation curve and p_50_ (the partial pressure of oxygen at which hemoglobin is 50% saturated with oxygen) under various conditions, including pre- and postexercise, as well as control and oxidative stress scenarios. Consistent with established literature [101], our results demonstrated a rightward shift in the oxygen dissociation curve following exercise. This shift enhances oxygen release from hemoglobin, thereby improving oxygen delivery to active muscles.

Of particular interest was the effect of oxidative stress on the oxygen dissociation curve. Postexercise p_50_ values were 35.40 mmHg under control conditions and 36.10 mmHg under oxidative stress conditions, indicating a slight rightward shift owing to oxidative stress. This shift suggests an increased ability of erythrocytes to release oxygen to working muscles, pointing to compromised muscle tissue oxygenation under oxidative stress. This observation is likely attributable to the 8.1% increase in 2,3-BPG levels under oxidative stress compared with the normal condition, which may also partially account for the reduced oxygenation and lower VO_2_peak observed during arm exercise under oxidative stress compared with the control condition, consistent with the known role of 2,3-BPG in modulating hemoglobin’s oxygen affinity [28, 31, 34].

Another potential mechanism by which oxidative stress could impair muscle oxygenation, and performance involves the increased formation of methemoglobin, which cannot transport oxygen. Although the interaction between time and condition was not statistically significant (*p* = 0.056), the effect of exercise on methemoglobin levels remains noteworthy. Oxidative stress increased methemoglobin levels by 17.8% compared with baseline pre-exercise, though this change was not statistically significant. Crucially, the physiologically relevant metric is the percentage increase of methemoglobin relative to total hemoglobin. At baseline, methemoglobin constituted 2.9% of total hemoglobin, rising to 3.4% postexercise, representing a 0.5 percentage point increase. While this increment likely has a minimal impact on oxygen-carrying capacity, oxidative stress may have induced the formation of other nonfunctional hemoglobin species, such as ferryl hemoglobin, which were not quantified in this study [102].

### Muscle Oxygenation

In an attempt to bridge the gap between the molecular and metabolic findings measured in erythrocytes and whole-body exercise performance, we assessed muscle oxygenation in working skeletal muscles using NIRS [67]. As expected, we observed a decrease in oxygenated hemoglobin (O_2_Hb) and an increase in deoxygenated hemoglobin (HHb) during both arm and leg exercises. These reciprocal changes indicate increased oxygen extraction by the working muscles, suggesting that the oxygen consumption rate of the muscles might exceed the delivery rate by erythrocytes.

The most important finding for the aims of our study; however, is that of a lower increase in deoxygenated HHb levels during exercise and during the first 3 min of recovery in the oxidative stress condition. The attenuated increase in HHb levels during exercise and recovery under oxidative stress implies that the muscles were less able to extract oxygen from the blood, suggesting a slower reoxygenation of the muscles. This is indicative of a less efficient delivery and usage of oxygen by the working muscles during oxidative stress. This muscle impairment to get the oxygen amount they need is likely partly the result of having erythrocytes less able to load and/or release the appropriate amount of oxygen owing to both impaired membrane integrity and bioenergetics crisis, which is also in line with our findings on reduced glycolytic flux. In summary, the impaired increase in deoxygenated HHb levels during exercise and recovery under oxidative stress, together with the reduced glycolytic flux and VO_2_peak, suggests that oxidative stress may compromise the efficiency of oxygen delivery and utilization by the muscles, ultimately impacting overall exercise performance.

### Exercise Performance

We assessed exercise performance by measuring leg and arm isometric, concentric, and eccentric peak torque as well as leg and arm VO_2_peak through incremental endurance tests. As expected, strength performance decreased in the eccentrically contracted leg, and VO_2_peak decreased by 8.4% during leg exercise under oxidative stress. Our findings align with existing literature, which shows that exercise-induced muscle damage reduces oxygenation and performance in the affected muscle group owing to impairments in metabolism, microcirculation, and neuromuscular function [103–105].

To our knowledge, studies on the interplay between eccentric contractions and oxidative stress have focused on the affected muscle group and few studies on the potential cross-over effect on other muscles [106]. These studies, while valuable, do not address the potential biochemical and systemic factors causing distant effects of exercise. In our study, 2 days after leg eccentric contractions, VO_2_peak decreased by 4.0% during the arm-crank test under oxidative stress. This suggests a systemic role for erythrocytes since the arm muscles were not directly affected by the single-leg protocol, relying on erythrocytes for oxygen transport. Given that total erythrocyte number and hemoglobin concentration remained unchanged (Supplementary Table S1), eccentric contraction-induced oxidative stress likely impaired oxygen transport and delivery by affecting erythrocyte metabolism and function. This aligns with the absence of oxidative stress effects on muscle strength performance, likely reflecting that these tests do not depend on the oxygen transport and release capacity of erythrocytes.

In exercise physiology, it is well-known that muscle energy metabolism determines muscle function and whole-body performance [107, 108]. At the onset of maximal exercise, ATP turnover rate increases by approximately 250-fold, and glycolytic flux by about 100-fold to meet muscle metabolic demands [109]. Our study adds that during maximal exercise, erythrocyte glycolytic flux can increase up to threefold. The most supported cause of endurance fatigue is a mismatch between energy supply and demand. In addition, reactive oxygen and nitrogen species are controlling exercise responses (e.g., contractile function, glucose uptake, vasodilation, and bioenergetics) [110]. Specific redox modifications, such as *S*-glutathionylation of proteins, contribute to exercise-induced muscle fatigue [111–114]. An optimal redox state is essential for muscle function, with both excessively reduced or oxidized states negatively impacting performance, as illustrated by the classic biphasic model [94, 115].

This optimal redox state is likely crucial for erythrocytes as well. Studies using generic oxidative stress biomarkers or targeted approaches have verified the oxidation of important redox-sensitive proteins in erythrocytes during exercise, such as peroxiredoxin 2, protein phosphatase 2, and GAPDH [116, 117]. Our findings suggest that oxidative stress in erythrocytes, sustained for 2 days following eccentric contractions, contributes to fatigue by reducing erythrocyte efficiency. It remains unclear, and cannot be determined from our study design, whether an energy crisis induced a redox crisis, a redox crisis led to an energy crisis, or if both occurred independently, impairing erythrocyte function.

Several nutritional interventions (e.g., carbohydrate, creatine, caffeine) have successfully enhanced substrate availability, improving endurance performance [118–120]. However, most studies focus exclusively on the bioenergetic and redox states of muscles, often neglecting the metabolic needs of other tissues or cells, such as erythrocytes. Our results indicate that oxidative stress in erythrocytes, which have limited repair capacity [121–124], might impair their function and contribute to endurance fatigue. Therefore, bioenergetic enhancers (e.g., pyruvate kinase activators; [30]) or redox enhancers (e.g., vitamin E; [125]), which have been used effectively in erythrocyte rejuvenation studies, may have ergogenic potential worth testing.

### Limitations

1. Most of our biochemical measurements are static (e.g., concentrations, activities). Incorporating more flux measurements would have better captured the dynamic nature of erythrocytes. For instance, while we observed significant changes in glycolytic flux, we did not measure possible changes in the flux of branching pathways.
2. We did not perform -omics analyses or other systems biology measurements, which could have provided a more comprehensive understanding of the molecular changes occurring in erythrocytes.
3. Most of our redox measurements were generic. Focusing on redox signaling and specific redox modifications would have provided deeper insights into the role of redox biology in erythrocytes.
4. We did not investigate the potential off-target effects of eccentric contraction-induced oxidative stress on other cells or tissues, such as the vascular endothelium or the skeletal muscles in the arms. Evidence of oxidative stress in leg muscles and its absence in arm muscles through biopsies would have further validated our experimental model (single-leg exercise and arm assessment) and strengthened the systemic role of erythrocytes.
5. Many factors that we did not measure (e.g., carbon monoxide, ferryl hemoglobin, and ATP levels) may have influenced hemoglobin binding properties and/or erythrocyte deformability.
6. We focused primarily on redox and energy changes, neglecting other aspects of erythrocyte biology, such as purine/nucleoside metabolism or biomechanical properties.
7. We did not measure the erythrocyte oxygen-carrying capacity and hemoglobin oxygen dissociation curve ex vivo. Instead, we computed the hemoglobin oxygen dissociation curve using previously published mathematical equations.

## Conclusions

The deceptively simple nature of erythrocytes has likely contributed to the general belief among nonspecialists in exercise physiology that they are merely passive oxygen carriers. Our evidence shows that erythrocytes, like any other cell in the human body, can respond to exercise and that erythrocyte metabolism is not just an “internal” biochemical matter but is crucial for controlling one of the most important biological processes: loading, transporting, and delivering oxygen. We do not claim to have identified the cause of endurance fatigue. However, our study highlights the need to focus on erythrocyte biology, which may add an overlooked cell to the list of contributors to fatigue and deepen our understanding of endurance physiology.

## Supplementary Information

Below is the link to the electronic supplementary material.

Supplementary file1 (DOCX 1316 KB)

Supplementary file2 (XLSX 17 KB)

## Declarations

### Availability of Data and Material

The data that support the findings of this study are openly available on GitHub: https://github.com/PanosChatzi/erythrocyte_study_statistical_analyses

### Code Availability

The code that supports the findings of this study are openly available on GitHub: https://github.com/PanosChatzi/erythrocyte_study_statistical_analyses; https://github.com/PanosChatzi/erythrocyte-oxygen-metabolism.
