# Supplementary material for: Erythrocyte Glycolytic and Redox Metabolism Affects Muscle Oxygenation and Exercise Performance: A Randomized Double-Blind Crossover Study in Men
Source: Sports Med. 2025 Jul 28;55(12):3157–81. doi: 10.1007/s40279-025-02279-2 (PMC12628466; doi:10.1007/s40279-025-02279-2)

**Journal:** Sports Medicine

**Erythrocyte glycolytic and redox metabolism affects muscle oxygenation and exercise performance: a randomized double-blind crossover study in men**

Panagiotis N. Chatzinikolaou^1*^, Nikos V. Margaritelis^1^, Vassilis Paschalis^2^, Anastasios A. Theodorou^3^, Eleni Moushi^3^, Ioannis S. Vrabas^1^, Antonios Kyparos^1^, Ioannis G. Fatouros^4^, Angelo D’Alessandro^5^ and Michalis G. Nikolaidis^1*^

^1^Department of Physical Education and Sports Science at Serres, Aristotle University of Thessaloniki, Serres, Greece

^2^School of Physical Education and Sport Science, National and Kapodistrian University of Athens, Athens, Greece

^3^Department of Life Sciences, School of Sciences, European University Cyprus, Nicosia, Cyprus

^4^Department of Physical Education and Sport Sciences, University of Thessaly, Trikala, Greece

^5^Department of Biochemistry and Molecular Genetics, University of Colorado Anschutz Medical Campus, Aurora, CO, USA

***Corresponding authors**

Panagiotis N. Chatzinikolaou

Department of Physical Education and Sports Science at Serres, Aristotle University of Thessaloniki, Serres, Greece

[chatzinpn@auth.gr](mailto:chatzinpn@auth.gr)

ORCiD: 0000-0002-8136-1638

Michalis G. Nikolaidis

Department of Physical Education and Sports Science at Serres, Aristotle University of Thessaloniki, Serres, Greece

[nikolaidis@auth.gr](mailto:nikolaidis@auth.gr)

ORCiD: 0000-0001-6165-8437

**SUPPLEMENTARY INFORMATION FILE 1**

Table of Contents

[Table S1. Baseline hematological profile 2](#_Toc201087641)

[Statistical analysis 2](#_Toc201087642)

[Figure S1. Arm vascular occlusion oxygenated (O_2_Hb), deoxygenated (HHb) and total hemoglobin (tHb) responses in the control (black circles) and oxidative stress condition (red squares). 8](#_Toc201087643)

[Figure S2. Leg vascular occlusion oxygenated (O_2_Hb), deoxygenated (HHb) and total hemoglobin (tHb) responses in the control (black circles) and oxidative stress condition (red squares). 9](#_Toc201087644)

[Figure S3. Blood pH levels in the control (black circles) and oxidative stress condition (red squares) at baseline, and two days later at pre-exercise and post-exercise (mean ± 95% CI). 10](#_Toc201087645)

## Table S1. Baseline hematological profile

| **Parameter** | **Control** | **Oxidative stress** |
| --- | --- | --- |
| Hematocrit (%) | 42.9 ± 3.5 | 43.4 ± 3.8 |
| Hemoglobin (g/L) | 149 ± 12 | 150 ± 13 |
| Erythrocytes (10^12^ cells) | 5.18 ± 0.27 | 5.15 ± 0.27 |
| White blood cells (10^9^ cells) | 6.82 ± 1.59 | 6.80 ± 1.66 |
| Platelets (10^9^ cells) | 238 ± 44 | 246 ± 36 |

Data are presented as mean ± SD.

## Statistical analysis

**Hematological profile**

Baseline total erythrocyte [t(19) = 0.95, p = 0.354, g = 0.204], white blood cell [W = 124, p = 0.498, rb = 0.181] and platelet count [t(19) = -0.76, p = 0.457, g = -0.67] was not different between the control and oxidative stress condition (Table S1). Hemoglobin concentration [W = 107.5, p = 0.940, rb = 0.024] and hematocrit [W = 63.5, p = 0.343, rb = -0.257] were also not different between the conditions.

**Muscle damage**

*Arm and leg ROM and DOMS*

No significant main effect of condition [F(1, 19) = 0.441, p = 0.515, η²_p_ = 0.023] and time [F(1, 19) = 1.521, p = 0.233, η²_p_ = 0.074] were observed on arm ROM. A significant interaction was detected [F(1, 19) = 7.675, p = 0.012, η²_p_ = 0.288], but post-hoc tests didn’t reveal any differences in arm ROM. Even though there was a significant main effect of condition [F(1, 19) = 5.52, p = 0.030, η²_p_ = 0.225], time [F(1, 19) = 5.52, p = 0.030, η²_p_ = 0.225] and interaction [F(1, 19) = 5.52, p = 0.030, η²_p_ = 0.225] on arm DOMS, post-hoc tests did not reveal any differences. A significant main effect of time [F(1, 19) = 186, p < 0.001, η²_p_ = 0.908], condition [F(1, 19) = 138, p < 0.001, η²_p_ = 0.879] and interaction [F(1, 19) = 150, p < 0.001, η²_p_ = 0.888] were also found on leg ROM. Post-48 h, leg ROM in the oxidative stress condition was significantly lower compared to baseline (g = -4.48, p < 0.001). Moreover, there was a significant main effect of condition [F(1, 19) = 136.58, p < 0.001, η²_p_ = 0.878], time [F(1, 19) = 136.58, p < 0.001, η²_p_ = 0.878], and interaction [F(1, 19) = 136.58, p < 0.001, η²_p_ = 0.878] on leg DOMS. Two days after the eccentric protocol, DOMS of the exercised leg was increased compared to baseline (g = 5.02, p < 0.001).

*Hemolysis and creatine kinase*

The results showed a significant main effect of condition [F(1, 19) = 10.45, p = 0.004, η²_p_ = 0.355], time [F(2, 38) = 77.85, p < 0.001, η²_p_ = 0.804] and interaction [F(1.373, 26.09) = 24.80, p < 0.001, η²_p_ = 0.566] on plasma hemoglobin levels. Two days after the oxidative stress condition, plasma hemoglobin levels were increased (g = 1.45, p < 0.001) and remained increased after the arm exercise (g = 1.54, p < 0.001), compared to baseline. In the control condition, post-arm exercise plasma hemoglobin was increased compared to pre-exercise (g = 0.84, p < 0.01) and baseline (g = 0.90, p < 0.001). A significant main effect of both condition [F(1, 19) = 87.98, p < 0.001, η²_p_ = 0.822] and time [F(1.23, 23.34) = 86.73, p < 0.001, η²_p_ = 0.820], as well as interaction [F(1.22, 23.13) = 100.72, p < 0.001, η²_p_ = 0.841] were observed on creatine kinase levels. Compared to baseline, two days after the oxidative stress condition creatine kinase levels were significantly increased (g = 3.46, p < 0.001) and remained increased after the arm exercise (g = 3.80, p < 0.001), whereas no changes were found in the control condition.

**Glycolysis**

*Glycolytic flux*

The results revealed a significant main effect of time [F(2.15, 40.77) = 38.35, p < 0.001, η²_p_ = 0.670] on glycolytic flux, but no main effect of condition time [F(1, 19) = 0.00133, p = 0.971, η²_p_ = 0.000]. A significant condition by time interaction was observed [F(2.24, 42.59) = 4.61, p = 0.013, η²_p_ = 0.195]. Post-hoc pairwise comparisons revealed that, in the control condition, glycolytic flux was found to increase immediately and 10 min after the arm exercise, compared to pre-exercise (g = 2.76, p < 0.0001 and g = 1.31, p < 0.001, respectively), and it was decreased 10 min (g = -0.73, p < 0.001) and 30 min post-exercise (g = -2.18, p < 0.001) compared to immediately post-exercise. In the oxidative stress condition, glycolytic flux was found increased immediately after the arm exercise compared to pre-exercise (g = 1.48, p = 0.0037), and 30 min post-exercise glycolytic flux was decreased compared to immediately post (g = -1.65, p = 0.0018) and 10-min post exercise (g = -0.95, p = 0.0037). Moreover, in the oxidative stress condition, glycolytic flux was higher compared to the control at the pre-exercise resting measurement (g = 0.62, p = 0.0279).

*Glycolytic flux under glucose and hydrogen peroxide challenges*

On the glucose challenge, there was a significant main effect of condition [F(1, 19) = 8.05, p = 0.011, η²_p_ = 0.298] and time [F(3, 57) = 6.070, p = 0.001, η²_p_ = 0.242] on glycolytic flux, but no interaction was observed [F(3, 57) = 0.256, p = 0.857, η²_p_ = 0.013]. On the hydrogen peroxide challenge, there was a significant main effect of condition [F(1, 19) = 5.33, p = 0.032, η²_p_ = 0.219] and time [F(4, 76) = 12.28, p < 0.001, η²_p_ = 0.393] on glycolytic flux, but no interaction [F(4, 76) = 0.825, p = 0.513, η²_p_ = 0.042].

*Hexokinase, phosphofructokinase, GAPDH and G6PD*

There was a significant condition by time interaction [F(2, 38) = 7.297, p = 0.002, η²_p_ = 0.277] and a main effect of time [F(2, 38) = 24.40, p < 0.001, η²_p_ = 0.562] on hexokinase activity, but no main effect of condition [F(1, 19) = 0.320, p = 0.578, η²_p_ = 0.017]. Post-hoc tests revealed that, in the control condition, arm exercise increased hexokinase activity compared to pre-exercise (g = 0.58, p = 0.0390) and baseline levels (g = 0.55, p = 0.011). The activity of hexokinase increased two days after the oxidative stress condition (g = 0.65, p = 0.0059) and after the arm exercise it remained increased compared to baseline (g = 0.80, p< 0.001). On phosphofructokinase activity, there was a significant main effect of time [F(1.90, 36.02) = 7.360, p = 0.002, η²_p_ = 0.279], but no effect of condition [F(1, 19) = 0.430, p = 0.521, η²_p_ = 0.022] or interaction [F(2, 38) = 0.0725, p = 0.930, η²_p_ = 0.004]. No significant main effects of condition [F(1, 19) = 0.283, p = 0.601, η²_p_ = 0.015] and interaction [F(2, 38) = 0.65, p = 0.530, η²_p_ = 0.033] were observed on GAPDH activity. However, there was a significant main effect of time [F(1.31, 24.89) = 4.14, p = 0.043, η²_p_ = .179]. On G6PD activity, the results revealed a significant main effect of time [F(1.19, 22.61) = 90.14, p < 0.001, η²_p_ = 0.826] and interaction [F(1.62, 30.71) = 4.13, p = 0.033, η²_p_ = 0.179]. No main effect of condition was detected [F(1, 19) = 0.355, p = 0.558, η²_p_ = 0.018]. Two days after the oxidative stress condition, G6PD activity was increased compared to baseline (g = 0.62, p<0.001). In both the control and oxidative stress conditions, G6PD activity was increased after the arm exercise compared to pre-exercise (g = 1.27, p < 0.001 and g = 0.90, p = 0.0011, respectively).

*2,3-BPG and methemoglobin*

There was a significant main effect of time [F(1.25, 23.83) = 55.56, p < 0.001, η²_p_ = 0.745] on 2,3-BPG concentration, but no main effect of condition [F(1,19) = 1.99, p = 0.175, η²_p_ = 0.095] and interaction [F(1.39, 26.42) = 3.06, p = 0.079 , η²_p_ = 0.139]. There was a significant main effect of time [F(2, 38) = 9.344, p < 0.001, η²_p_ = 0.330] on methemoglobin concentration, but no main effect of condition [F(1, 19) = 0.022, p = 0.883, η²_p_ = 0.001] or interaction [F(1.389, 26.395) = 3.595, p = 0.056, η²_p_ = 0.159].

**Redox metabolism**

*F_2_-isoprostanes, protein carbonyls and glutathione*

The results revealed a significant main effect of condition [F(1, 19) = 9.25, p = 0.007, η²_p_ = 0.328] and time [F(1.42, 27.04) = 39.91, p < 0.001, η²_p_ = 0.677] on the levels of F_2_-isoprostanes within erythrocytes. Moreover, there was a significant condition by time interaction [F(1.47, 27.98) = 4.72, p = 0.026, η²_p_ = 0.199]. Post-hoc tests revealed that post-exercise F_2_-isoprostanes levels were higher compared to pre-exercise and baseline in both the control (g = 0.77, p < 0.001 and g = 0.83, p = 0.018, respectively) and oxidative stress conditions (g = 0.52, p = 0.029 and g = 1.51, p < 0.001). Moreover, two days after the oxidative stress condition, F_2_-isoprostanes were increased compared to baseline (g = 0.98, p = 0.009) and compared to the control pre-exercise value (g = 1.00, p = 0.046). A significant main effect of time [F(2, 38) = 20.15, p < 0.001, η²_p_ = 0.515] and interaction [F(1.38, 26.15) = 7.75, p = 0.005, η²_p_ = 0.290] were also observed on the protein carbonyls levels within erythrocytes. There was no significant main effect of condition [F(1, 19) = 1.96, p = 0.178, η²_p_ = 0.093]. Protein carbonyl levels were increased after the arm exercise compared to pre-exercise (g = 0.76, p = 0.007) in the control condition. Compared to baseline, protein carbonyl levels were higher two days after the oxidative stress condition (g = 0.86, p = 0.008) and remained higher at post-exercise (g = 1.02, p = 0.007). The analysis showed a significant main effect of time [F(2, 38) = 58.71, p < 0.001, η²_p_ = 0.756] and interaction [F(2, 38) = 13.83, p < 0.001, η²_p_ = 0.421] on the concentration of glutathione in erythrocytes. A trend towards a main effect of condition [F(1, 19) = 4.24, p = 0.053, η²_p_ = 0.183] was observed but did not reach statistical significance. In the control condition, post-exercise glutathione concentration was significantly lower compared to pre-exercise (g = -1.30, p < 0.001) and baseline (g = -1.21, p < 0.001). In the oxidative stress condition, baseline glutathione concentration was significantly lower than pre-exercise (g = -0.81, p < 0.001) and post-exercise (g = -1.15, p < 0.001) concentrations. Additionally, the pre-exercise glutathione was lower in the oxidative stress compared to the control condition (g = 0.93, p < 0.001).

*Superoxide dismutase, catalase, glutathione peroxidase and glutathione reductase*

The analysis revealed a significant main effect of time [F(1, 19) = 13.35, p = 0.002, η²_p_ = 0.413], condition [F(2, 38) = 11.10, p < 0.001, η²_p_ = 0.369] and a condition by time interaction [F(2, 38) = 13.72, p < 0.001, η²_p_ = 0.419] on superoxide dismutase activity. Compared to baseline, superoxide dismutase activity was found increased two days after the oxidative stress condition (g = 0.89, p = 0.0259) and remained increased post-exercise (g = 1.18, p = 0.0012). Similarly, a significant main effect of time [F(1, 19) = 25.95, p = 0.002, η²_p_ = 0.577], condition [F(2, 38) = 28.40, p < 0.001, η²_p_ = 0.599] and interaction [F(2, 38) = 12.04, p < 0.001, η²_p_ = 0.388] were observed on catalase activity. Catalase activity was found increased two days after the oxidative stress condition (g = 1.05, p = 0.0016), and remained increased at post-exercise compared to baseline (g = 1.46, p < 0.001). In the control condition, arm exercise increased catalase activity (g = 0.49, p = 0.013). Pre-exercise catalase activity was higher in the oxidative condition compared to control at pre-exercise (g = 1.12, p = 0.0013) and post-exercise (g = 1.03, p < 0.001). Moreover, there was also a significant main effect of condition [F(1, 19) = 4.47, p = 0.048, η²_p_ = 0.190] and time [F(2, 38) = 23.39, p < 0.001, η²_p_ = 0.552], as well as interaction [F(2, 38) = 32.47, p < 0.001, η²_p_ = 0.631] on glutathione peroxidase activity. In the control condition, glutathione peroxidase activity was higher post-exercise compared to pre-exercise (g = 0.43, p = 0.023) and baseline (g = 0.33, p = 0.003). There was an increase in glutathione peroxidase activity two days after the oxidative stress condition (g = 1.39, p < 0.001) and remained increased post-exercise (g = 0.89, p < 0.001) compared to baseline. Additionally, two days after the oxidative stress condition, it was found higher compared to pre-exercise control (g = 1.44, p = 0.0042). On glutathione reductase activity, a significant main effect of time [F(2, 38) = 5.06, p = 0.011, η²_p_ = 0.21] was observed, but no main effect of condition [F(1, 19) = 0.0785, p = 0.782, η²_p_ = 0.004] or interaction [F(1.14, 21.73) = 0.56, p = 0.485, η²_p_ = 0.029].

*Vitamin C, vitamin E, NADH and NADPH*

Regarding the non-enzymatic redox molecules, the results revealed a significant main effect of time [F(1.65, 31.31) = 12.45, p < 0.001, η²_p_ = 0.396] on vitamin C concentration, but no main effect of condition [F(1, 19) = 0.003, p = 0.959, η²_p_ = 0.000] or interaction [F(2, 38) = 1.33, p = 0.277, η²_p_ = 0.065]. Moreover, there was a significant main effect of condition [F(1, 19) = 19.12, p < 0.001, η²_p_ = 0.502] and time [F(1.45, 27.47) = 7.01, p = 0.004, η²_p_ = 0.270] on Vitamin E concentration, but no interaction [F(1.37, 25.98) = 0.510, p = 0.538, η²_p_ = 0.026]. Regarding NADH and NADPH, there was no significant main effect of condition [F(1, 19) = 0.072, p = 0.791, η²_p_ = 0.004; and F(1, 19) = 4.27, p = 0.053, η²_p_ =0.184, respectively] or time [F(2, 38) = 2.846, p = 0.071, η²_p_ = 0.130; and F(2, 38) = 1.02, p = 0.370, η²_p_ = 0.051]. However, a significant interaction was observed in both NADH [F(1.34, 25.48) = 5.244, p = 0.022, η²_p_ = 0.216] and NADPH [F(1.50, 28.46) = 4.04, p = 0.039, η²_p_ = 0.175]. Compared to baseline, NADH was significantly decreased two days after the oxidative stress condition (g = 0.53, p < 0.001). Post-hoc tests did not show any differences in NADPH concentration.

**Exercise near-infrared spectroscopy**

*Arm oxygenation*

During the arm incremental test, a significant main effect of time [F(1.554, 29.531) = 144.31, p < 0.001, η²_p_ = 0.884] was observed on arm O_2_Hb levels, but no significant effect of condition [F(1, 19) = 0.19, p = 0.666, η²_p_ = 0.010] or interaction [F(4.955, 94.144) = 1.70, p = 0.142, η²_p_ = 0.082]. There was a significant main effect of time [F(1.853, 35.206) = 232.08, p < 0.001, η²_p_ = 0.924] and interaction [F(5.594, 106.283) = 2.406, p = 0.036, η²_p_ = 0.112] on HHb levels, but no main effect of condition [F(1, 19) = 1.13, p = 0.302, η²_p_ = 0.056]. In both conditions, arm HHb levels were progressively increased until exhaustion (p < 0.001) and returned to baseline levels after 3 min of recovery (p < 0.001). On tHb levels, there was a significant main effect of time [F(3.356, 63.767) = 26.132, p < 0.001, η²_p_ = 0.579], but no main effect of condition [F(1, 19 ) = 0.033, p = 0.859, η²_p_ = 0.002] or interaction [F(5.109, 97.066) = 0.419, p = 0.838, η²_p_ = 0.022].

*Leg oxygenation*

During the cycling incremental test, there was no significant main effect of condition [F(1, 19) = 1.30, p = 0.268, η²_p_ = 0.064] on leg O_2_Hb levels, but a significant main effect of time [F(2.407, 45.726) = 142.06, p < 0.001, η²_p_ = 0.882] and interaction [F(4.093, 77.769) = 5.65, p < 0.001, η²_p_ = 0.229] were observed. Leg O_2_Hb levels were significantly decreased from baseline until exercise exhaustion (p < 0.001) and returned to baseline levels after a 3 min recovery (p < 0.001). After 2 min recovery, O_2_Hb reached higher levels in the control compared to the oxidative stress condition (p = 0.019). Similarly, a significant main effect of time [F(2.699, 51.288) = 163.33, p < 0.001, η²_p_ = 0.896] and interaction [F(4.895, 92.997) = 5.86, p < 0.001, η²_p_ = 0.236] were found on HHb levels, but no main effect of condition [F(1, 19) = 0.008, p = 0.928, η²_p_ = 4.426 × 10^-4^]. In both conditions, leg HHb levels decreased during exercise onset (p < 0.001), then continued to increase until exhaustion (p < 0.001), and returned to baseline levels after 3 min recovery (p < 0.001). Finally, there was a significant main effect of time [F(3.109, 59.071) = 20.52, p < 0.001, η²_p_ = 0.519] and interaction [F(4.694, 89.191) = 3.2, p = 0.012, η²_p_ = 0.144] on tHb levels, but no main effect of condition [F(1, 19) = 0.756, p = 0.396, η²_p_ = 0.038]. In both conditions, at exercise initiation tHb levels were first decreased (p < 0.001) and then increased progressively until exhaustion (p < 0.001), and returned to baseline levels after 2 min recovery (p < 0.001).

**Vascular occlusion near-infrared spectroscopy**

*Arm occlusion*

Regarding the arm vascular occlusion tests, a main effect of time [F(1.500, 28.504) = 185.544, p < 0.001, η²_p_ = 0.907] on O_2_Hb was observed, but no effect of condition [F(1, 19) = 0.087, p = 0.772, η²_p_ = 0.005] or interaction [F(2.370, 45.032) = 2.65, p = 0.073, η²_p_ = 0.122]. There was a main effect of time [F(1.370, 26.035) = 212.95, p < 0.001, η²_p_ = 0.918] and interaction [F(3.289, 62.490) = 6.36, p < 0.001, η²_p_ = 0.251] on HHb, but no main effect of condition [F(1, 19) = 3.12, p = 0.093, η²_p_ = 0.141]. In both conditions, HHb was significantly decreased during the 5 min occlusion (p < 0.001) and recovered immediately upon reperfusion (p < 0.001). HHb at 4 min and 5 min of occlusion was significantly lower in the oxidative stress compared to the control condition (p < 0.001 and p = 0.012, respectively). A significant main effect of time [F(1.65, 31.44) = 4.84, p = 0.020, η²_p_ = 0.203] was observed on tHb, but no effect of condition [F(1, 19) = 0.39, p = 0.542, η²_p_ = 0.020] or interaction [F(3.36, 63.78) = 1.96, p = 0.122, η²_p_ = 0.094].

*Leg occlusion*

In the leg occlusion tests, a significant main effect of condition [F(1, 19) = 19.165, p < 0.001, η²_p_ = 0.502], time [F(2.004, 38.007) = 286.89, p < 0.001, η²_p_ = 0.938] and interaction [F(2.798, 53.170) = 15.65, p < 0.001, η²_p_ = 0.452] was observed on O_2_Hb. In both conditions, O_2_Hb was significantly decreased through the 5 min occlusion (p < 0.001) and reoxygenated to baseline levels at recovery (p < 0.001). At 2 min and 3 min of occlusion, O_2_Hb reached lower levels in the control compared to oxidative stress condition (p < 0.001), but reached higher levels at 1 min recovery (p = 0.005). On HHb, there was a significant main effect of condition [F(1, 19) = 15.08, p < 0.001, η²_p_ = 0.443] and time [F(1.630, 30.965) = 139.30, p < 0.001, η²_p_ = 0.880] as well as interaction [F(5.026, 95.503) = 22.01, p < 0.001, η²_p_ = 0.537]. In both conditions, HHb levels increased throughout the occlusion (p < 0.001) and returned to baseline levels at recovery (p < 0.001). HHb levels in the oxidative stress condition were significantly lower compared to the control condition after 3, 4 and 5 min of occlusion (p < 0.001). Finally, a main effect of time [F(1.985, 37.711) = 48.92, p < 0.001, η²_p_ = 0.720] and interaction [F(3.85, 73.12) = 8.87, p < 0.001, η²_p_ = 0.318] were detected on tHb levels, but no effect of condition [F(1, 19) = 0.168, p = 0.687, η²_p_ = 0.009]. After 5 min occlusion, tHb levels were higher in the control compared to the oxidative stress condition (p = 0.005).

**Isokinetic dynamometry performance**

Regarding the arm isokinetic dynamometry, there was no main effect of condition or time on the peak isometric [F(1, 19) = 1.196, p = 0.288, η²_p_ = 0.059; and F(1, 19) = 0.161, p = 0.692, η²_p_ = 0.008, respectively], concentric [F(1, 19) = 3.926, p = 0.062, η²_p_ = 0.171; and F(1, 19) = 0.594, p = 0.450, η²_p_ = 0.030] and eccentric torque [F(1, 19) = 0.095, p = 0.762, η²_p_ = 0.005; and F(1, 19) = 0.112, p = 0.741, η²_p_ = 0.006]. There was a significant interaction on the arm isometric [F(1, 19) = 9.559, p = 0.006, η²_p_ = 0.335] and eccentric peak torque [F(1, 19) = 10.868, p = 0.004, η²_p_ = 0.364], but not on concentric peak torque [F(1, 19) = 4.118, p = 0.057, η²_p_ = 0.178]. Post-hoc did not reveal any significant differences.

Regarding the leg isometric, concentric and eccentric peak torque, there was a significant main effect of condition [F(1, 19) = 98.7, p < 0.001, η²_p_ = 0.839; F(1, 19) = 109.5, p < 0.001, η²_p_ = 0.852; and F(1, 19) = 92.4, p < 0.001, η²_p_ =0.830, respectively] and time [F(1, 19) = 141.2, p < 0.001, η²_p_ = 0.881; F(1, 19) = 137.0, p < 0.001, η²_p_ = 0.878; and F(1, 19) = 111.7, p < 0.001, η²_p_ =0.855], as well as interaction [F(1, 19) = 142.4, p < 0.001, η²_p_ = 0.882; F(1, 19) = 61.7, p < 0.001, η²_p_ = 0.765; and F(1, 19) = 131.9, p < 0.001, η²_p_ = 0.874]. In the oxidative stress condition, two days after the eccentric protocol, the peak torque was significantly decreased compared to baseline in the isometric (g = -1.99, p < 0.001), concentric (g = 1.45, p < 0.001) and eccentric protocol (p = 2.38, p < 0.001). No changes were observed in the control condition.

**Peak oxygen uptake**

In the oxidative stress condition, the arm VO_2_peak was significantly lower compared to the control condition [t(19) = -2.446, p = 0.024, g(rm) = -0.163], even though the oxidative stress was induced by performing exercise with the leg. A Wilcoxon signed-rank test was performed on leg VO_2_peak, which was found to be lower in the oxidative stress condition compared to control [W = 25, p = 0.00169, rb = -0.668], probably due to the muscle damage in the leg. In the arm incremental test, peak RER did not differ between the two conditions [t(19) = 1.955, p = 0.065, g = 0.208], but it was significantly lower in the oxidative stress compared to the control condition in the cycling incremental test [W = 9, p = 0.004, rb = -0.621]. Peak heart rate values during the arm and leg incremental tests were not different between the two conditions [t(19) = -0.046, p = 0.9638, g = 0.009; and [t(19) = 0.818, p = 0.4233, g = 0.111, respectively].

## Figure S1. Arm vascular occlusion oxygenated (O_2_Hb), deoxygenated (HHb) and total hemoglobin (tHb) responses in the control (black circles) and oxidative stress condition (red squares).


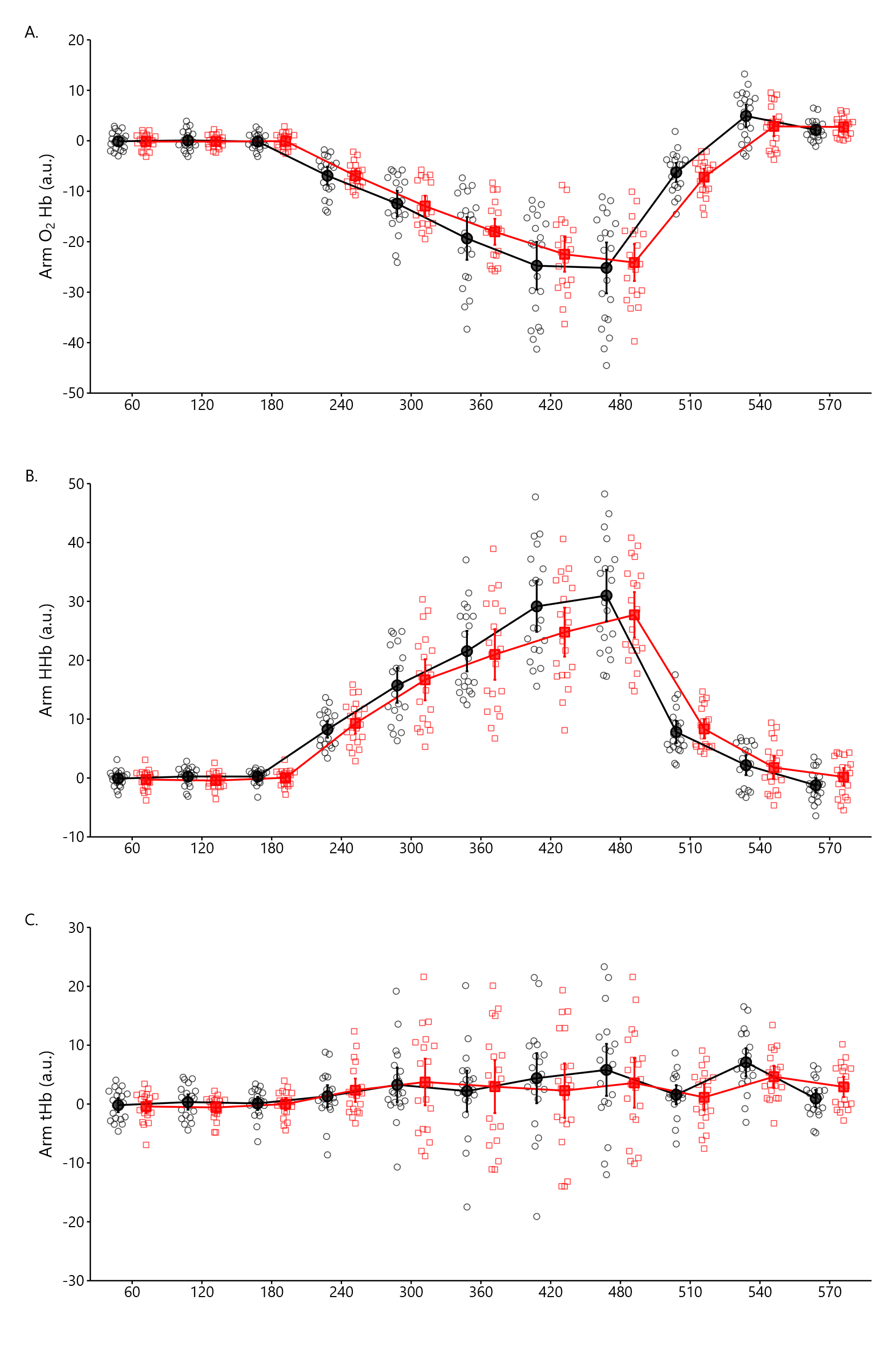


## Figure S2. Leg vascular occlusion oxygenated (O_2_Hb), deoxygenated (HHb) and total hemoglobin (tHb) responses in the control (black circles) and oxidative stress condition (red squares).


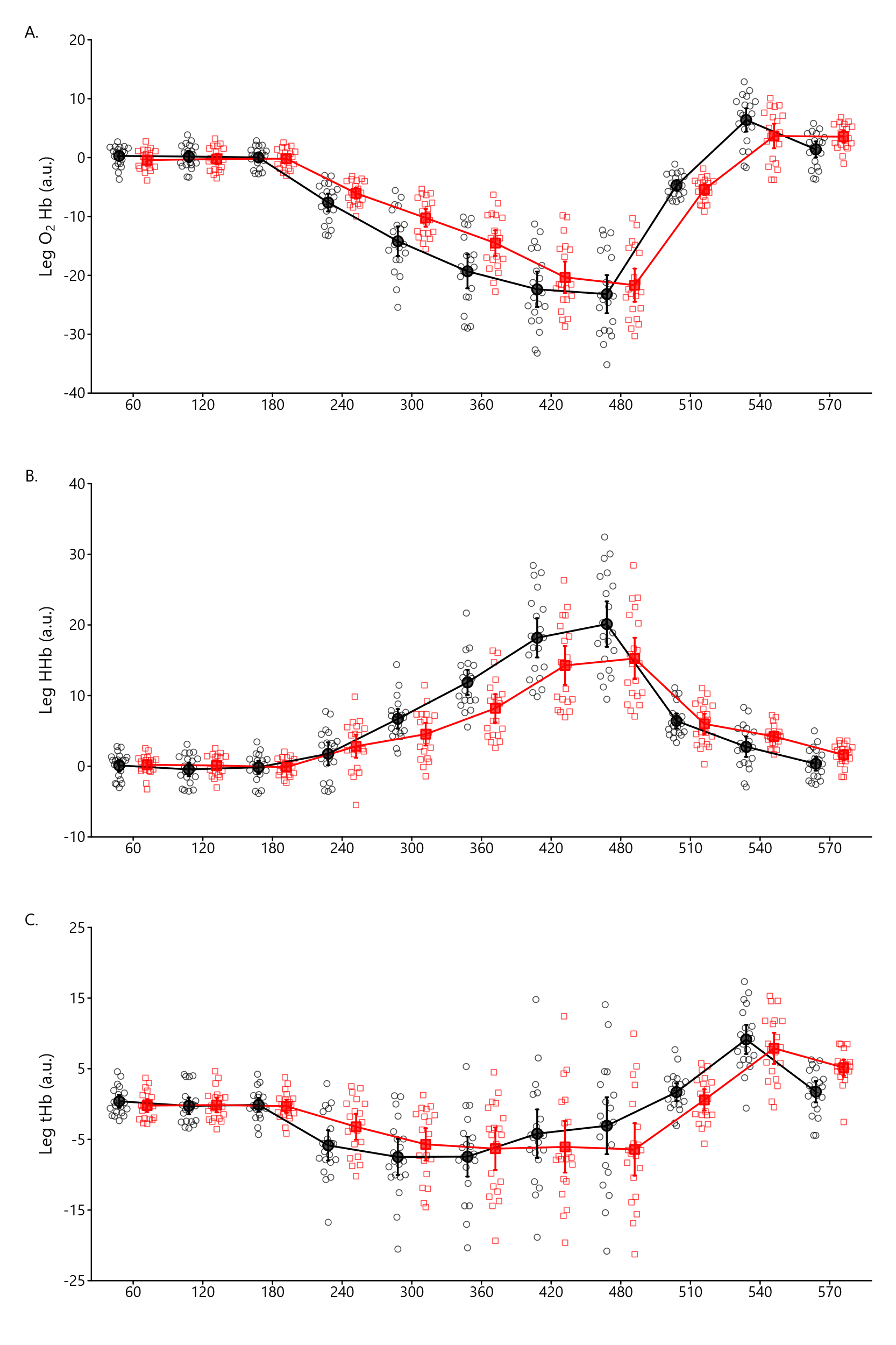


## Figure S3. Blood pH levels in the control (black circles) and oxidative stress condition (red squares) at baseline, and two days later at pre-exercise and post-exercise (mean ± 95% CI).


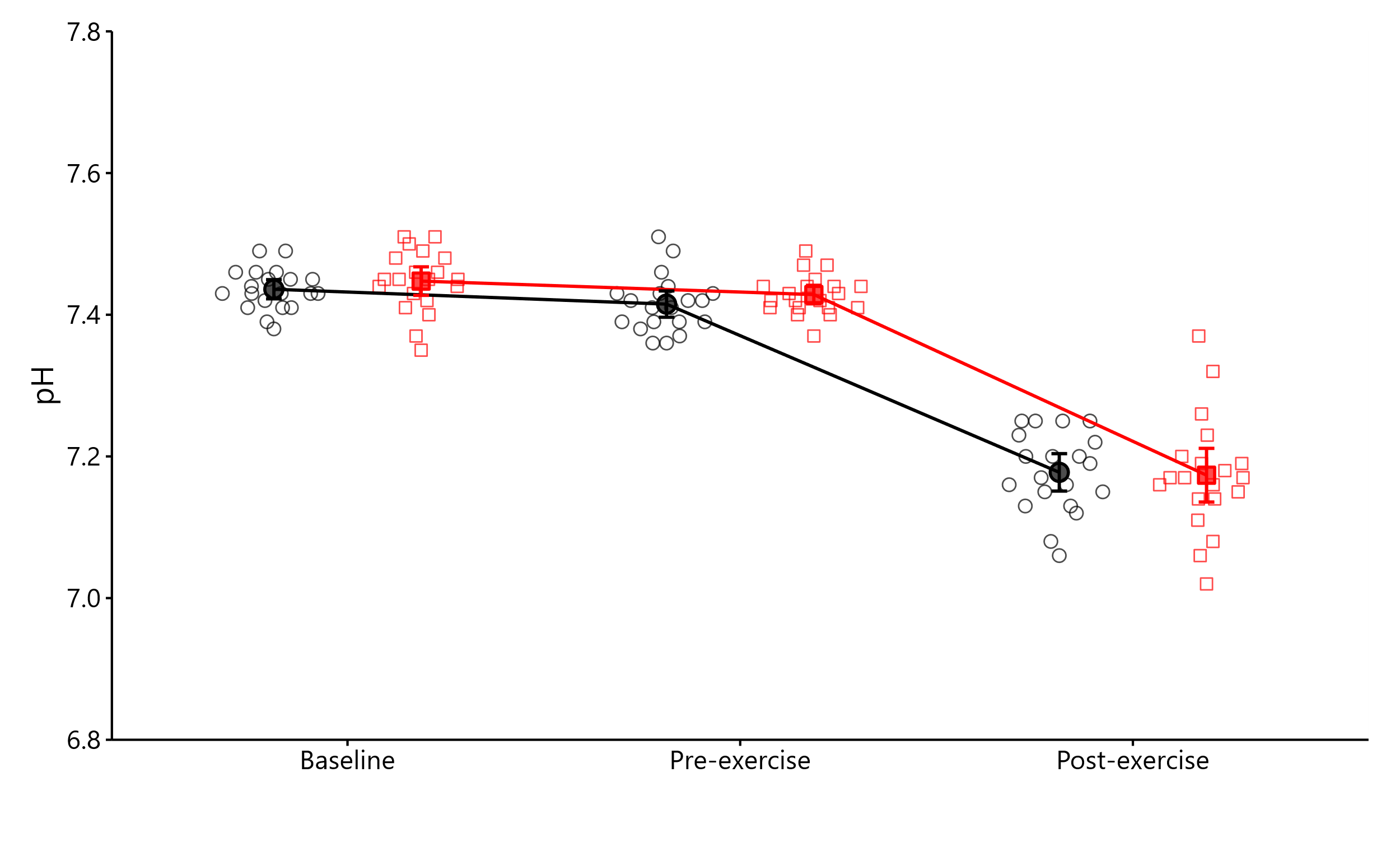

Supplement: Supplementary file 1 — Supplementary file1 (DOCX 1316 KB) [file 40279_2025_2279_MOESM1_ESM.docx]
